# Detection of high-risk carbapenem-resistant *Klebsiella pneumoniae* and *Enterobacter cloacae* isolates using volatile molecular profiles

**DOI:** 10.1038/s41598-018-31543-x

**Published:** 2018-09-05

**Authors:** Christiaan A. Rees, Mavra Nasir, Agnieszka Smolinska, Alexa E. Lewis, Katherine R. Kane, Shannon E. Kossmann, Orkan Sezer, Paola C. Zucchi, Yohei Doi, Elizabeth B. Hirsch, Jane E. Hill

**Affiliations:** 1 Geisel School of Medicine, Dartmouth College, Hanover, NH 03755 United States; 2 Department of Pharmacology and Toxicology, Maastricht University Medical Centre, Maastricht, 6200 MD The Netherlands; 3 Dartmouth College, Hanover, NH 03755 United States; 4 Department of Molecular Biology and Microbiology, Tufts University, Boston, MA 02111 United States; 5 Division of Infectious Diseases, School of Medicine, University of Pittsburgh, Pittsburgh, PA 15213 United States; 6 College of Pharmacy, University of Minnesota, Minneapolis, MN 55455 United States; 7 Thayer School of Engineering, Dartmouth College, Hanover, NH 03755 United States

## Abstract

Infections caused by carbapenem-resistant *Enterobacteriaceae* (CRE) are alarming in the clinical setting, as CRE isolates often exhibit resistance to most clinically-available antibiotics. *Klebsiella pneumoniae* carbapenemase (KPC) is the most common carbapenemase carried by CRE in North America and Europe, frequently detected in isolates of *K*. *pneumoniae*, *Escherichia coli*, and *Enterobacter cloacae*. Notably, KPC-expressing strains often arise from clonal lineages, with sequence type 258 (ST258) representing the dominant lineage in *K*. *pneumoniae*, ST131 in *E*. *coli*, and ST78 and ST171 in *E*. *cloacae*. Prior studies have demonstrated that carbapenem-resistant *K*. *pneumoniae* differs from carbapenem-susceptible *K*. *pneumoniae* at both the transcriptomic and soluble metabolomic levels. In the present study, we sought to determine whether carbapenem-resistant and carbapenem-susceptible isolates of *K*. *pneumoniae*, *E*. *coli*, and *E*. *cloacae* produce distinct volatile metabolic profiles. We were able to identify a volatile metabolic fingerprint that could discriminate between CRE and non-CRE with an area under the receiver operating characteristic curve (AUROC) as high as 0.912. Species-specific AUROCs were as high as 0.988 for *K*. *pneumoniae* and 1.000 for *E*. *cloacae*. Paradoxically, curing of KPC-expressing plasmids from a subset of *K*. *pneumoniae* isolates further accentuated the metabolic differences observed between ST258 and non-ST258.

## Introduction

Carbapenem-resistant *Enterobacteriaceae* (CRE) represent one of the most substantial threats to public health since the beginning of the antibiotic era. The emergence of isolates resistant to most, if not all, available antimicrobial agents has led to substantial morbidity, mortality, and healthcare-associated costs^1,2^. Recent estimates suggest that attributable mortality may be as high as 44%, particularly in the setting of bacteremia^1^, with total economic costs exceeding $553 million annually in the United States based on current incidence^3^. Since their emergence in the late 1980s, CRE have been identified on all inhabited continents, and the incidence of infections caused by these organisms has increased steadily in the first two decades of the 21^st^ century^4,5^. Presently, *Klebsiella pneumoniae* carbapenemase (KPC) is the most commonly-encountered carbapenemase in both North America and Europe^6^.

There are three primary mechanisms of carbapenem resistance in *Enterobacteriaceae*: 1) enzymatic degradation of carbapenem antibiotics via the production of carbapenemases, 2) reduced accessibility of carbapenems to the periplasmic space via mutations in outer membrane porins, and 3) increased carbapenem export via augmented expression of efflux pump components^7^. Although multiple mechanisms of resistance may exist concurrently in a single isolate (*e*.*g*., a porin mutation coupled with expression of an extended-spectrum β-lactamase, which alone is not sufficient for resistance)^8^, infections caused by carbapenemase-producing isolates result in substantially higher overall mortality relative to non-carbapenemase-producing isolates^9^. While the mechanism(s) responsible for this excess mortality have not yet been elucidated, it is likely that other characteristics of carbapenemase-expressing strains, such as increased virulence, may contribute. For example, *Klebsiella pneumoniae* isolates belonging to sequence type 258 (ST258), the dominant carbapenemase-expressing lineage in much of North America and Europe^10^, inhibits phagocytosis by human neutrophils; a concurrent phenotype seemingly unrelated to carbapenemase expression itself^11^.

The association of clonal bacterial lineages with specific antimicrobial resistance patterns is not unique to carbapenem resistance in *K*. *pneumoniae*; it has been widely reported across a range of species and resistance patterns, including vancomycin resistance in *Enterococcus faecium*^12^, and methicillin resistance in *Staphylococcus aureus*^13^. Commonly-encountered carbapenemase-producing lineages in *Enterobacteriaceae* include ST258 in *K*. *pneumoniae*, ST131 in *Escherichia coli*^14^, and ST78 and ST171 in *Enterobacter cloacae*^15,16^. Of interest, transcriptomic profiling of *K*. *pneumoniae* has revealed distinct profiles associated with carbapenemase-producing ST258 isolates compared with carbapenem-susceptible isolates belonging to other lineages, even with respect to genes that have no apparent relationship to carbapenem resistance^17^. Indeed, Bruchmann and colleagues demonstrated that nearly 1,200 unique transcripts were differentially abundant between ST258 and non-ST258 isolates of *K*. *pneumoniae*, associated with a wide range of functions including carbohydrate metabolism, nucleotide metabolism, and response to cellular stress. It is plausible that other carbapenem-resistant lineages of *Enterobacteriaceae* also differ metabolically from their carbapenem-susceptible relatives, although this has not been evaluated to-date.

In the present study, we profile bacterially-derived volatile metabolites produced *in vitro* for the purposes of: (1) discriminating between *Klebsiella pneumoniae* carbapenemase (KPC)-producing and non-carbapenemase-producing *Enterobacteriaceae* (CPE and non-CPE, respectively), (2) identifying a high risk clonal lineage of *K*. *pneumoniae* (ST258), and (3) assessing the influence of multidrug resistance plasmids on the volatile molecular signature of *K*. *pneumoniae* ST258 isolates. Using three distinct machine learning algorithms (partial least squares-discriminant analysis, random forest, and support vector machines), we were able to discriminate between CPE and non-CPE, and identify a metabolic fingerprint associated with the main carbapenemase-expressing *K*. *pneumoniae* lineage, ST258. In addition, through the analysis of cured ST258 strains, we were able to demonstrate that the volatile metabolic fingerprint associated with CPE likely includes contributions from both the bacterial chromosome as well as extrachromosomal elements. The present findings suggest that CPE and non-CPE differ metabolically from one another, and these metabolic differences could represent potential diagnostic and/or therapeutic targets in the detection and treatment of infections caused by these multidrug-resistant pathogens.

## Results

### Volatile metabolic fingerprints distinguish CPE from non-CPE

We hypothesized that volatile metabolic fingerprints could distinguish CPE from non-CPE, and measured the volatile metabolites produced *in vitro* by both *bla*_KPC_-positive and *bla*_KPC_-negative clinical isolates of *K*. *pneumoniae*, *E*. *coli*, and *E*. *cloacae*. The isolates used in the present study (*n* = 117) included 60 *K*. *pneumoniae* (28 *bla*_KPC_-positive and 32 *bla*_KPC_-negative), 37 *E*. *coli* (19 *bla*_KPC_-positive and 18 *bla*_KPC_-negative), and 20 *E*. *cloacae* (10 *bla*_KPC_-positive and 10 *bla*_KPC_-negative) (Supplementary Fig. S1). In general, *bla*_KPC_-positive isolates belonged to relatively few clonal lineages, with dominant sequence types (STs) including ST258 for *K*. *pneumoniae* (68%), ST171 for *E*. *cloacae* (100%), and ST131 for *E*. *coli* (63%). In contrast, *bla*_KPC_-negative isolates represented a much broader range of STs across all three species, with 47 distinct STs represented (1.3 isolates per ST) (Supplementary Figs S2–S4). Less heterogeneity was observed amongst *bla*_KPC_-negative *E*. *coli* relative to either *K*. *pneumoniae* or *E*. *cloacae*, with five isolates belonging to ST131 (28%), three belonging to ST69 (17%), and three belonging to ST95 (17%). It is noteworthy that ST131 represented the dominant ST for both carbapenemase-producing (CP) and non-carbapenemase-producing (non-CP) *E*. *coli*, as this is in contrast to both *K*. *pneumoniae* and *E*. *cloacae*, for which the dominant ST amongst CP isolates was not represented at all amongst non-CP isolates. This may be due to the inclusion of multidrug-resistant (but carbapenem-susceptible) isolates of *E*. *coli* within our non-CP population, as ST131 is strongly associated with other patterns of antibiotic resistance, such as resistance to fluoroquinolones or extended-spectrum cephalosporins^18^.

*Enterobacteriaceae* isolates were grown to early stationary phase in Mueller-Hinton broth, which occurred at approximately 12 h post-inoculation. The bacterial cell density (as determined via the optical density at 600 nm (OD_600_)) differed between CP and non-CP *K*. *pneumoniae* at the time of harvesting (mean OD_600_: 2.33 vs. 2.38, *p* = 0.005), but did not differ between CP and non-CP isolates of either *E*. *coli* (2.32 vs. 2.32, *p* = 0.76) or *E*. *cloacae* (2.46 vs. 2.47, *p* = 0.71). Volatile metabolites were analyzed using headspace solid-phase microextraction two-dimensional gas chromatography time-of-flight mass spectrometry (HS-SPME-GC × GC-TOFMS), and we identified 169 volatile metabolites that were produced by one or more isolates from all three species which we define as the “core metabolome.” We employed three machine learning algorithms (random forest (RF), partial least-squares discriminant analysis (PLS-DA), and linear support vector machines (linear SVM)) to identify a subset of these metabolites that were important for discriminating between CPE and non-CPE. Additional details about these algorithms are provided in the Materials and Methods section.

**Figure 1:**
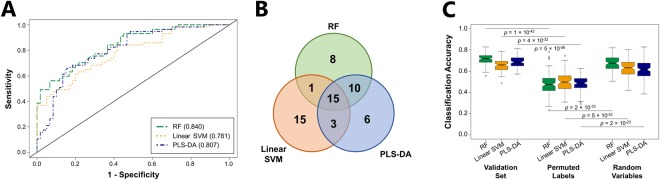
Volatile metabolic fingerprints distinguish CPE from non-CPE. (**A**) ROC curves for the discrimination between CPE and non-CPE generated using class probabilities for validation set samples. Green: RF; Orange: linear SVM; Blue: PLS-DA. Values in parentheses indicate AUROC. (**B**) Venn diagram depicting overlap of metabolites between RF (green), linear SVM (orange), and PLS-DA (blue) for the top 34 discriminatory volatile metabolites across all 100 discovery-validation splits. (**C**) Box plots depicting classification accuracies from RF (green), linear SVM (orange), and PLS-DA (blue) for validation set samples using: (1) the top 34 discriminatory metabolites (left), (2) the top 34 discriminatory metabolites with permuted sample class labels (center), and (3) 34 randomly-selected metabolites with correct sample class labels (right).

Metabolites were ranked according to their discriminatory ability for the comparison of CPE and non-CPE. Initially utilizing the top 20% of discriminatory features (*n* = 34), we obtained an optimal area under the receiver operating characteristic curve (AUROC) for validation set samples of 0.840 using RF, with an optimal sensitivity of 0.702 and specificity of 0.783 (Fig. 1A, Supplementary Table S1). PLS-DA and linear SVM yielded similar but slightly lower AUROCs of 0.807 and 0.781, respectively. Reducing the number of metabolites included in the RF model from 34 to 20 resulted in no reduction in AUROC (0.840), and further reduction to only five metabolites resulted in an AUROC of 0.801 (Supplementary Fig. S5). These top discriminatory metabolites were identified as 2-phenylacetate, 2-nonanone, one aldehyde, and two 2-ketones (Table 1). The inclusion of more than 34 metabolites did not improve classification accuracy for linear SVM, PLS-DA, or RF.

**Table 1:** Discriminatory volatile metabolites of the core metabolome.

Putative Identification	Class	RI	Match Score	Variable Importance Rank				DA
				RF	Linear SVM	PLS-DA	Overall	
***More abundant in CPE cultures***								
2-phenylethyl acetate	EST	1316	827	**1**	**3**	**1**	**1.5**	***
*Unknown hydrocarbon*	CH	1338	—	**39**	**82**	116	78.8	10.86
*Unknown 2-ketone*	KET	1342	—	52	**50**	61	54.2	8.42
*Unknown hydrocarbon*	CH	1282	—	**46**	113	57	72.1	5.12
*Unknown hydrocarbon*	CH	1116	—	**38**	77	**19**	44.4	4.30
*Unknown ester*	EST	902	—	**24**	116	**22**	54.0	2.54
*Unknown 2-ketone*	KET	1545	—	**9**	**18**	**6**	**10.9**	1.98
*Unknown hydrocarbon*	CH	1059	—	79	**49**	82	70.2	1.74
*Unknown hydrocarbon*	CH	1014	—	**28**	82	**19**	42.8	1.74
*Unknown hydrocarbon*	CH	1205	—	97	**59**	93	83.1	1.50
Benzene	BEN	701	843	112	**67**	110	96.2	1.50
2-methylpentane	CH	†	878	78	**63**	**36**	59.2	1.28
2-methyl-1-pentene	CH	†	892	64	93	**33**	63.3	1.24
*Unknown ester*	EST	1457	—	74	111	**41**	75.4	1.18
*p*-xylene	BEN	896	804	74	75	**45**	64.9	1.14
*Unknown benzene derivative*	BEN	996	—	**46**	**60**	63	56.3	1.06
*Unknown sulfur-containing*	S-C	809	—	**14**	98	113	74.9	1.03
***More abundant in non-CPE cultures***								
2-methyl-2-butanol	ALC	684	906	57	**47**	85	62.6	0.98
*Unknown sulfur-containing*	S-C	809	—	63	**45**	91	66.3	0.98
2-decanone	KET	1241	823	**19**	**22**	**23**	**21.2**	0.95
2,6,6-trimethylbicyclo[3.1.1]hept-2-ene	CH	949	862	134	**68**	97	99.4	0.95
*Unknown 2-ketone*	KET	1222	—	91	**46**	123	86.9	0.94
Methylpyrazine	HET	859	886	81	**62**	119	87.4	0.93
*Unknown 2-ketone*	KET	1035	—	**6**	**56**	**9**	**23.6**	0.93
Tetradecane	CH	1403	802	**31**	98	90	73.2	0.93
*Unknown 2-ketone*	KET	933	—	**11**	**68**	**34**	**37.5**	0.90
Benzaldehyde	ALD	1024	962	55	69	**36**	53.4	0.85
Propanal	ALD	†	855	47	**45**	54	48.6	0.83
2-nonanone	KET	1138	874	**6**	**51**	**19**	**25.5**	0.82
*Unknown aldehyde*	ALD	942	—	**25**	84	**38**	48.8	0.81
Methyl acetate	EST	†	895	**37**	75	81	64.4	0.81
2-methyl-3-isopropylpyrazine	HET	1086	837	**39**	101	90	76.6	0.79
4-methylene-1-(1-methylethyl)-bicyclo[3.1.0]hexane	CH	999	890	**28**	**49**	**28**	**35.0**	0.76
2-methyl-1-propanol	ALC	678	813	69	**61**	**46**	58.7	0.75
1-methyl-4-(1-methylethenyl)-cyclohexene	CH	1048	867	**12**	**13**	**12**	**12.6**	0.71
2,5-dimethylpyrazine	HET	946	944	**31**	78	**34**	47.9	0.70
2-propenal	ALD	†	916	**34**	**49**	**23**	**35.1**	0.70
2-methylpropanal	ALD	†	957	**17**	110	**10**	45.5	0.70
3-methylbutanal	ALD	693	934	**24**	**45**	**22**	**30.6**	0.69
*Unknown aldehyde*	ALD	1149	—	**7**	**46**	**16**	**22.9**	0.68
2-methylbutanal	ALD	701	882	**18**	77	**9**	33.6	0.62
2,3-pentanedione	KET	735	928	**23**	85	**9**	38.7	0.62
3-ethyl-2,5-dimethylpyrazine	HET	1114	877	**12**	**46**	**20**	**26.0**	0.32

Only metabolites for which a putative compound identification or compound class assignment could be determined are presented. RI: Experimentally-determined retention index; DA: Differential abundance (average compound abundance in CPE cultures divided by average compound abundance in non-CPE cultures); ALC: alcohol; ALD: aldehyde; BEN: benzene-derivative; CH: hydrocarbon; EST: ester; HET: heterocycle; KET: ketone; S-C: sulfur-containing; ^†^Retention indices less than 600 not extrapolated; —: Match score not provided for compounds without putative identifications; ^***^Not detected in non-CPE cultures. Bolded feature ranks indicate that the metabolite was included amongst the top 20% most highly discriminatory metabolites for that algorithm.

Because the three machine learning algorithms employed in this study utilize different approaches for the identification of discriminatory features, we sought to compare the selection of discriminatory metabolites across RF, linear SVM, and PLS-DA. When we compare the three models with respect to the 34 most highly discriminatory metabolites selected, we find that 15 metabolites were common to RF, PLS-DA, and linear SVM, 14 were selected by two of three, and 29 were selected by only one (Fig. 1B). RF and PLS-DA were most similar in their selection of discriminatory metabolites, as defined by the coefficient of correlation between feature ranks for the two algorithms (*r* = 0.64, *p* = 6.1 × 10^−8^), while the feature ranks for metabolites selected by linear SVM did not significantly correlate with those of either RF or PLS-DA (Supplementary Fig. S6). Despite differences in the composition and relative importance of the metabolites selected by each algorithm, overall sample classification accuracy was similar for RF, linear SVM, and PLS-DA (0.716, 0.658, and 0.690, respectively) (Fig. 1C). To ensure that our models were not over-fit, we permuted sample labels (*i*.*e*., randomly designated isolates in the discovery set as either “CPE” or “non-CPE”), which resulted in accuracies of 0.513, 0.508, and 0.511 for RF, linear SVM, and PLS-DA, respectively, approximating random probability. In contrast, the selection of 34 metabolites at random yielded classification accuracies of 0.674, 0.629, and 0.611 for RF, linear SVM, and PLS-DA, respectively, suggesting that CPE and non-CPE differ extensively across their volatile metabolic profiles.

**Figure 2:**
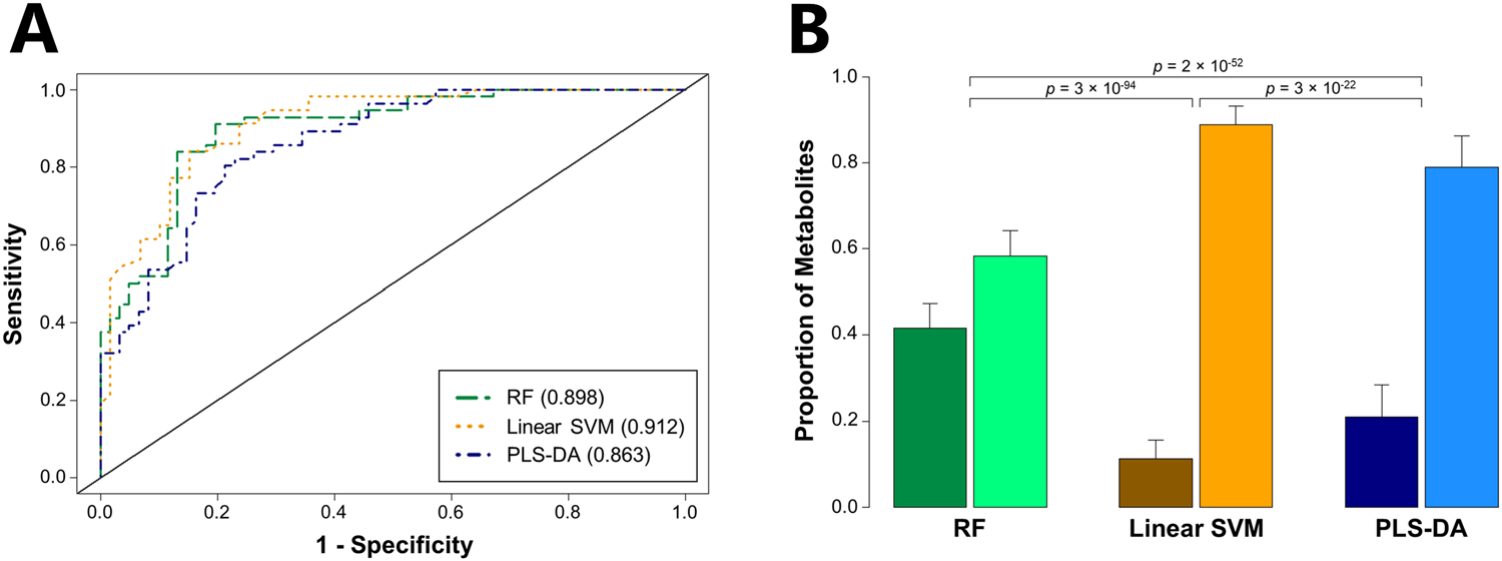
Analysis of the pan metabolome improves classification accuracy for distinguishing CPE from non-CPE. (**A**) ROC curves for the discrimination between CPE and non-CPE generated using class probabilities for validation set samples. Green: RF; Orange: linear SVM; Blue: PLS-DA. Values in parentheses indicate AUROC. (**B**) Bar plot depicting the proportion of discriminatory metabolites (amongst the top 34 most discriminatory metabolites) derived from either the core metabolome (left bar in each pair) or pan metabolome (right bar in each pair). Green: RF; orange: linear SVM; blue: PLS-DA.

We also sought to determine whether we could improve accuracy for distinguishing CPE from non-CPE through the analysis of all 891 volatile metabolites produced by one or more isolates from *any* of the three species (the “pan metabolome”), rather than *all* three species (the “core metabolome”). For consistency with our previous analysis of the core metabolome, we included the same number of metabolites (*n* = 34) in our analysis of the pan metabolome. Modest increases in AUROC were observed for all three algorithms, with the largest observed for linear SVM (from 0.781 to 0.912) (Fig. 2A), which was also the top-performing algorithm overall. Reducing the number of metabolites included in the linear SVM model from 34 to 15 resulted in only a minimal decrease in AUROC (from 0.912 to 0.908, Supplementary Fig. S5), while further reduction to only 5 metabolites resulted in an AUROC of 0.864. These top discriminatory metabolites included one metabolite from the core metabolome (2-phenylacetate), and four from the pan metabolome (one ester, and three unknown metabolites) (Tables 1 and 2). The substantial improvement of linear SVM may be due, in part, to the selection of fewer core metabolites (11%) as discriminatory relative to either PLS-DA (21%) or RF (42%) (Fig. 2B).

**Table 2:** Discriminatory volatile metabolites of the pan metabolome (excluding core metabolites).

Putative Identification	Class	RI	Match Score	Variable Importance Rank				Species	DA^‡^
				RF	Linear SVM	PLS-DA	Overall		
***More abundant in CPE cultures***									
2,3,3-trimethylpentane	CH	738	934	210	342	**32**	194.6	*Kp*	***
*Unknown hydrocarbon*	CH	843	—	215	361	**38**	204.4	*Kp*	***
*Unknown hydrocarbon*	CH	875	—	207	236	**37**	160.1	*Kp*	***
*Unknown benzene derivative*	BEN	1055	—	208	284	**36**	175.9	*Kp*	***
*Unknown hydrocarbon*	CH	1510	—	213	331	**31**	191.5	*Kp*	***
*Unknown hydrocarbon*	CH	1516	—	210	333	**32**	191.6	*Kp*	***
*Unknown hydrocarbon*	CH	1555	—	212	347	**36**	198.2	*Kp*	***
*Unknown ester*	EST	1081	—	**9**	**13**	**7**	**9.7**	*Kp*, *Eco*	33.79
*Unknown hydrocarbon*	CH	994	—	**9**	**20**	**6**	**11.5**	*Kp*, *Eco*	24.80
*Unknown hydrocarbon*	CH	859	—	**52**	208	**15**	91.5	*Kp*	20.63
4-methyloctane	CH	865	900	**26**	587	**20**	210.9	*Kp*, *Eco*	14.12
*Unknown hydrocarbon*	CH	819	—	89	181	**24**	98.0	*Kp*	8.58
*Unknown ester*	EST	747	—	**33**	135	**40**	69.3	*Kp*	6.18
*Unknown hydrocarbon*	CH	1222	—	302	**69**	190	186.9	*Ecl*	5.83
*Unknown 4-ketone*	KET	1013	892	237	**68**	218	174.1	*Kp*, *Eco*	1.78
*Unknown alcohol*	ALC	815	—	239	**90**	189	172.3	*Ecl*	1.26
***More abundant in non-CPE cultures***									
Octanal	ALD	1046	—	**21**	220	134	125.1	*Kp*, *Eco*	0.80
Hexanal	ALD	839	836	**16**	**108**	**21**	**48.3**	*Kp*, *Eco*	0.62
*Unknown alcohol*	ALC	1116	—	**43**	186	**52**	93.6	*Kp*, *Eco*	0.56
*Unknown S-containing*	S-C	1048	—	224	**102**	397	241.1	*Eco*	0.25
*Unknown N-containing*	N-C	911	—	**11**	**34**	**3**	**16.0**	*Kp*	0.18
Ethylcyclohexane	CH	844	835	**28**	**24**	**20**	**23.9**	*Kp*, *Eco*	0.05
*Unknown N-containing*	N-C	989	—	**37**	**49**	**9**	**31.3**	*Kp*	0.00
*Unknown alcohol*	ALC	1196	—	260	**72**	132	154.9	*Ecl*	0.00

RI: Experimentally-determined retention index; DA: Differential abundance (average compound abundance in CPE cultures divided by average compound abundance in non-CPE cultures); ALC: alcohol; ALD: aldehyde; BEN: benzene derivative; CH: hydrocarbon; EST: ester; KET: ketone; N-C: nitrogen-containing; S-C: sulfur-containing;—: Match score not provided for compounds without putative identifications; *Kp*: *K. pneumoniae*; *Eco*: *E. coli*; *Ecl*: *E. cloacae*; ^‡^calculated using only the species observed to produce the metabolite; ***Not detected in non CPE cultures. Bolded feature ranks indicate that the metabolite was included amongst the top 20% most highly discriminatory metabolites for that algorithm.

Having demonstrated that we could broadly discriminate between CPE and non-CPE using both the core and pan metabolome, we sought to determine whether our ability to distinguish CP from non-CP isolates differed across species. For the core metabolome, which yielded an optimal AUROC of 0.840, classification accuracies from RF were highest for isolates of *K*. *pneumoniae* (0.883), followed by *E*. *cloacae* (0.850), and *E*. *coli* (0.405) (Supplementary Fig. S7). For the pan metabolome, which yielded an optimal AUROC of 0.912, classification accuracies from linear SVM were highest for *E*. *cloacae* (0.950), followed by *K*. *pneumoniae* (0.933) and *E*. *coli* (0.649) (Supplementary Fig. S8). Taken together, our findings indicate that the inclusion of species-specific volatile metabolites of the pan metabolome modestly improves statistical model performance relative to the analysis of the core metabolome, with the most dramatic improvement observed for *E*. *coli* (+0.244), followed by *E*. *cloacae* (+0.100) and *K*. *pneumoniae* (+0.050).

Of the 106 metabolites identified as discriminatory using RF, PLS-DA, or linear SVM in our analysis of both the core and pan metabolomes, putative identifications and/or compound class assignments could be assigned to 67 (63%). Table 1 encompasses metabolites of the core metabolome (*n* = 43), while Table 2 includes additional metabolites belonging to the pan metabolome (*n* = 24). Thirty-three of these 67 most discriminatory metabolites (49%) were more abundant in CPE cultures, while the remaining 34 (51%) were more abundant in non-CPE cultures, indicating that both groups produced metabolites with discriminatory ability. Of note, the CPE-associated metabolites were enriched for: benzene derivatives (100% of all benzene derivatives were more abundant in CPE cultures), esters (83%), and hydrocarbons (79%). Non-CPE-associated metabolites were enriched for: aldehydes (100%), heterocycles (100%), alcohols (80%), and ketones (67%). In bacteria, different volatile metabolites belonging to a particular compound class often arise from a common metabolic process (*e*.*g*., 2-ketones arising from the metabolism of fatty acids)^19^. Therefore, the propensity of specific molecular classes to be more abundant in one group relative to the other supports the existence of fundamental biological differences between CPE and non-CPE that are reflected in their volatile metabolomes.

### Species-specific fingerprinting improves discrimination between carbapenemase-producing and non-carbapenemase-producing isolates of *K*. *pneumoniae* and *E*. *cloacae*, but not *E*. *coli*

For the diagnosis of bacterial infections, species-level identification nearly always precedes determination of antibiotic susceptibilities. Therefore, we sought to determine whether we could improve classification accuracy through the analysis of each species individually. We first confirmed our ability to discriminate between isolates of *K*. *pneumoniae*, *E*. *cloacae*, and *E*. *coli* at the species level using volatile metabolic signatures (100% classification accuracy using RF, data not shown), a finding consistent with previous *in vitro* data^20,21^. We then sought to identify species-specific volatile metabolic signatures that could distinguish CP from non-CP isolates of *K*. *pneumoniae*, *E*. *cloacae*, and *E*. *coli*.

**Figure 3:**
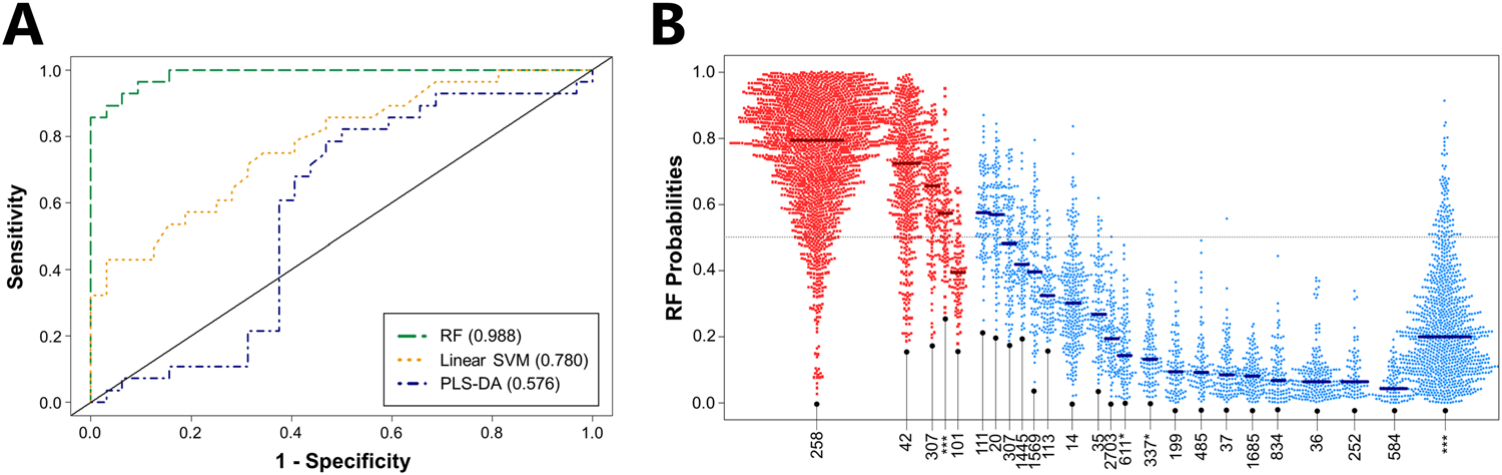
Volatile metabolic fingerprints discriminate between CP and non-CP *K*. *pneumoniae*. (**A**) ROC curves for the discrimination between CP and non-CP *K*. *pneumoniae* generated using class probabilities for validation set samples. Green: RF; Orange: linear SVM; Blue: PLS-DA. Values in parentheses indicate AUROC. (**B**) Bee swarm plot depicting class probabilities (*i*.*e*., CP versus non-CP) for *K*. *pneumoniae* isolates, divided by ST. Red: CP *K*. *pneumoniae*; blue: non-CP *K*. *pneumoniae*. Points towards the top of the plot indicate a higher probability of classifying as CP, while those towards the bottom of the plot indicate a higher probability of classifying as non-CP. Horizontal bars represent the median class probability for each ST. ^***^: Previously unreported ST.

First, we evaluated the ability of volatile metabolic fingerprints to discriminate between CP (*n* = 28) and non-CP (*n* = 32) *K*. *pneumoniae*, using the top 34 discriminatory metabolites identified for the comparison of these two groups, and obtained a nearly-perfect AUROC of 0.988 using RF (the best-performing algorithm), with an optimal sensitivity of 0.929 and specificity of 0.938 (Fig. 3A, Supplementary Table S1). Reducing the number of metabolites included in the RF model from 34 to 10 results in only a minimal decrease in AUROC (from 0.988 to 0.983), while further reduction to only 5 metabolites results in an AUROC of 0.970 (Supplementary Fig. S5). These top discriminatory metabolites included two from the core metabolome (one unknown aldehyde and one molecule for which neither a putative identification nor compound class assignment could be determined), and three from the pan-metabolome (octanal, one unknown N-containing molecule, and one unknown ester).

Amongst the CP isolates, those belonging to ST258, which represents the dominant *bla*_KPC_-expressing ST in the United States and Europe^22^ as well as in our collection of CP isolates (*n* = 19), classified correctly most often according to RF class probabilities (median = 0.79) (Fig. 3B). Importantly, CP isolates belonging to ST42 (*n* = 5), ST307 (*n* = 1), and one previously unreported ST (*n* = 1) also classified correctly, indicating that the volatile metabolic fingerprint that we report is capable of identifying CP isolates across a range of STs, rather than just those belonging to the single, dominant ST258 lineage (Fig. 3B). In addition, 29 of 31 non-CP STs (including 10 previously unreported STs) classified correctly, with only ST111 (*n* = 1) and ST20 (*n* = 1) misclassifying as CP. Taken together, these findings suggest that volatile metabolic fingerprints can discriminate between CP and non-CP *K*. *pneumoniae* across a broad range of STs, including isolates belonging to the dominant ST258 lineage.

**Figure 4:**
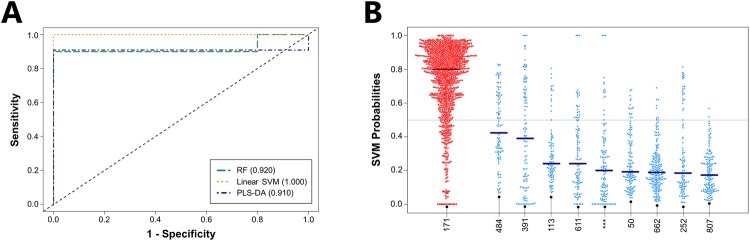
Volatile metabolic fingerprints discriminate between CP and non-CP *E*. *cloacae*. (**A**) ROC curves for the discrimination between CP and non-CP *E*. *cloacae* generated using class probabilities for validation set samples. Green: RF; Orange: linear SVM; Blue: PLS-DA. Values in parentheses indicate AUROC. (**B**) Bee swarm plot depicting class probabilities (*i*.*e*., CP versus non-CP) for *E*. *cloacae* isolates, divided by ST. Red: CP *E*. *cloacae*; blue: non-CP *E*. *cloacae*. Points towards the top of the plot indicate a higher probability of classifying as CP, while those towards the bottom of the plot indicate a higher probability of classifying as non-CP. Horizontal bars represent the median class probability for each ST. ^***^: Previously unreported ST.

We next analyzed the volatile metabolic fingerprints produced by CP (*n* = 10) and non-CP (*n* = 10) *E*. *cloacae* isolates, with all CP isolates belonging to a single ST, ST171. Again using the same number of metabolites as in previous comparisons (*n* = 34), we were able to discriminate between the two groups using linear SVM, which was the best-performing algorithm, with an associated AUROC of 1.000 (Fig. 4A, Supplementary Table S1). Reducing the number of metabolites included in the linear SVM model from 34 to only five did not substantially reduce the AUROC (1.000 to 0.990) (Supplementary Fig. S5). Of the top five most discriminatory metabolites, three belonged to the core metabolome (2-phenylacetate, tetradecane, and one sulfur-containing molecule), and two to the pan metabolome (one ester and one unknown compound). Class probabilities calculated as a function of ST demonstrated clear differences between the CP isolates, which all belonged to ST171 (median = 0.80), and the non-CP isolates belonging to nine distinct STs (median = 0.23) (Fig. 4B). Because CP *E*. *cloacae* outbreaks are uncommon relative to either CP *K*. *pneumoniae* or CP *E*. *coli*, dominant CP clonal lineages are less well-defined. The ST171 isolates used in the present study, however, represents an outbreak strain from the northern United States between 2011 and 2012, derived from a single clonal lineage^23^.

We also analyzed the volatile metabolites produced by CP (*n* = 19) and non-CP (*n* = 18) isolates of *E*. *coli*, and again using the top 34 most discriminatory metabolites, obtained an AUROC of 0.626 from PLS-DA (the best-performing algorithm), with an optimal sensitivity of 0.700 and specificity of 0.529 (Supplementary Fig. S9, Supplementary Table S1). The PLS-DA class probabilities demonstrated that while CP isolates belonging to ST964, ST3866, and ST536, as well as non-CP isolates belonging to ST1193, ST10, and ST372 tended to classify correctly (class probabilities >0.600 or <0.400, respectively), the remaining nine STs did not (Supplementary Fig. S10). We note that ST131, which is the dominant ST for both the CP (*n* = 12, 63%) and non-CP isolates (*n* = 5, 28%) yielded a wide range of class probabilities (ranging from 0.00 to 1.00), and these class probabilities were not significantly different between CP and non-CP ST131 isolates (*p* = 0.82). Although misclassification of ST131 isolates was not the sole reason for the relatively poor classification accuracy obtained for *E*. *coli* isolates, the preponderance of isolates belonging to ST131 in both the CP and non-CP isolate collections was undoubtedly a major contributing factor.

### Volatile metabolic signatures identify *K*. *pneumoniae* isolates belonging to ST258

**Figure 5:**
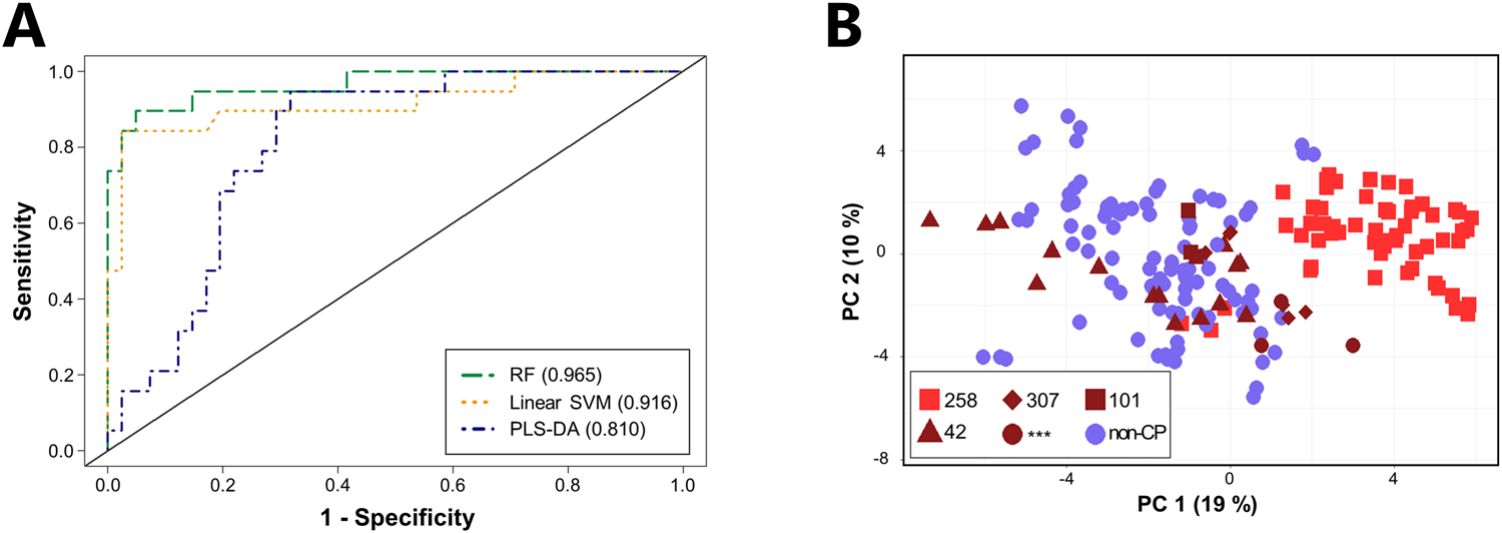
Volatile metabolic fingerprints discriminate between ST258 and non-ST258 isolates of *K*. *pneumoniae*. (**A**) ROC curves for the discrimination between ST258 and non-ST258 *K*. *pneumoniae* generated using class probabilities for validation set samples. Green: RF; Orange: linear SVM; Blue: PLS-DA. Values in parentheses indicate AUROC. (**B**) PC scores plot generated using the 52 discriminatory metabolites identified across RF, linear SVM, and PLS-DA. Red squares: ST258; dark red triangles: ST42; dark red diamonds: ST307; dark red circles: previously unreported ST; dark red squares: ST101; blue circles: non-CP isolates (all STs).

Given the propensity of CP *K*. *pneumoniae* isolates to arise from relatively few, successful clonal lineages, we assessed the ability of our approach to identify isolates belonging to the specific, high-risk clonal *K*. *pneumoniae* ST258 lineage, which accounts for between approximately 70–100% of carbapenem-resistant *K*. *pneumoniae* cases in the United States and Europe. ST258 isolates (*n* = 19) could be distinguished from non-ST258 isolates (*n* = 41, which included 33 other STs encompassing both CP and non-CP isolates), using the top 34 discriminatory metabolites, with an AUROC of 0.965 from RF (Fig. 5A). Reducing the number of metabolites included in the RF model from 34 to 10 does not influence the AUROC (remaining at 0.965), while further reduction to only 5 metabolites results in a modest decrease to 0.903 (Supplementary Fig. S5). Notably, only one of the five most highly discriminatory metabolites identified in the comparison of ST258 versus non-ST258 was also amongst the top five most highly discriminatory metabolites identified in the comparison of CP versus non-CP *K*. *pneumoniae* (an ester). Of the remaining four, two belonged to the core metabolome (a 2-ketone and an unknown metabolite), and two belonged to the pan metabolome (an ester and a hydrocarbon).

Fifty-two metabolites were identified as discriminatory between ST258 and non-ST258 across all three algorithms. Using these, we generated a principal component (PC) scores plot, which revealed that isolates belonging to ST258 formed a cluster away from both non-CP isolates, as well as away from CP isolates belonging to other STs (ST42, ST101, ST307, and the novel ST) (Fig. 5B). Non-ST258 CP isolates clustered amongst the non-CP isolates, suggesting that this metabolic signature is predominantly sequence type (rather than phenotype) specific. Taken together, these findings demonstrate that *K*. *pneumoniae* isolates belonging to ST258 produce a distinct volatile metabolic signature relative to both non-ST258 CP and non-CP isolates, suggesting the potential utility of a volatile metabolite-based approach for the identification of certain high-risk, clonal bacterial lineages. For the remaining *bla*_KPC_-positive *K*. *pneumoniae* STs, there were not enough isolates (between one and five per ST) to identify ST-associated volatile metabolic signature, although it is plausible that other *K*. *pneumoniae* lineages may have distinct metabolic signatures as well.

We were unable to identify a volatile metabolic signature that could differentiate *E*. *coli* ST131 (irrespective of carbapenem susceptibility) from other *E*. *coli* STs (including both CP and non-CP isolates). For all three machine learning algorithms, AUROCs were less than 0.500, indicating that volatile metabolic signatures could not distinguish *E*. *coli* ST131 from other *E*. *coli* STs (Supplementary Fig. S11, Supplementary Table S1).

### Cured *K*. *pneumoniae* ST258 exhibit a distinct volatile molecular profile from wild-type (*bla*_KPC_-positive) ST258

**Figure 6:**
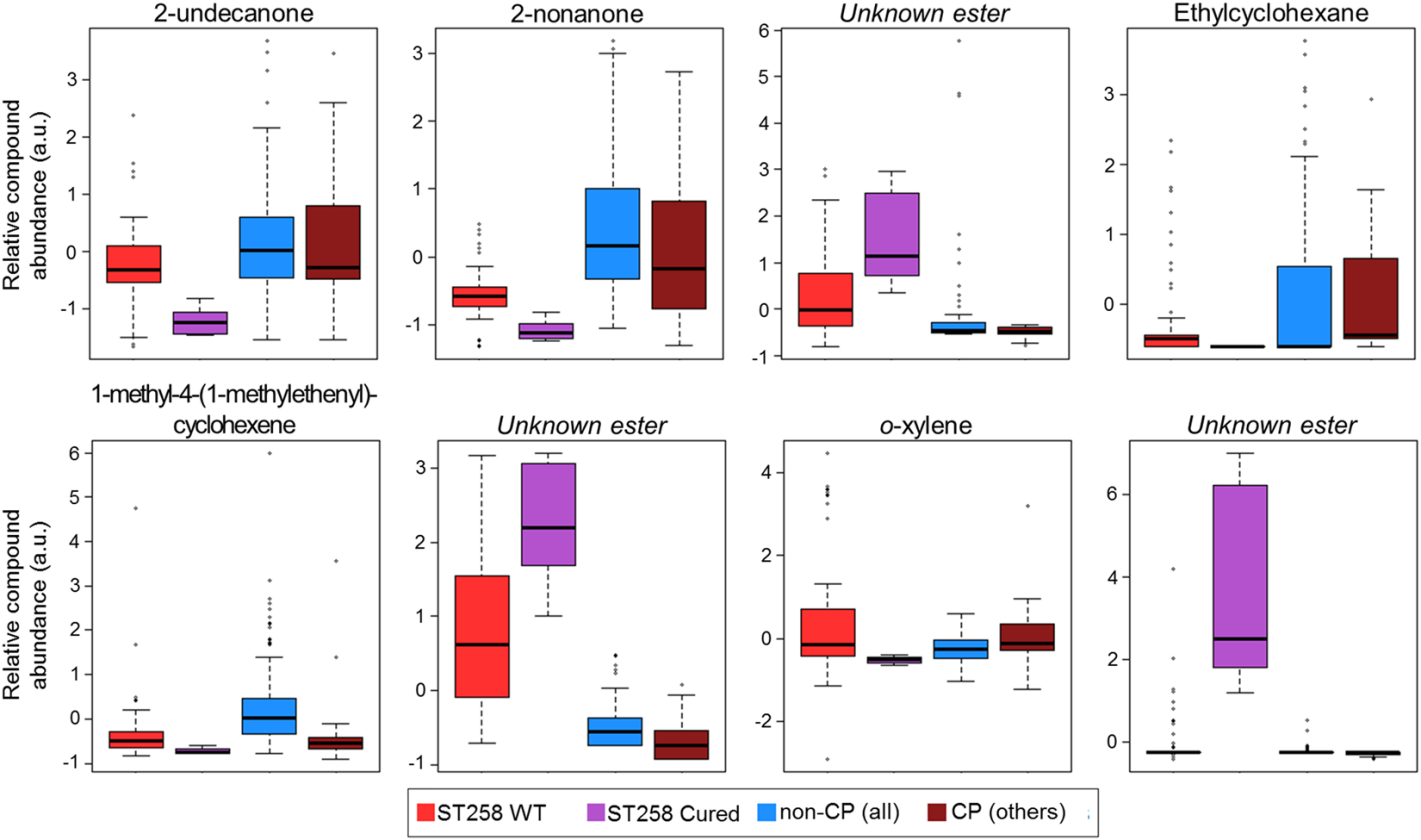
Plasmid curing alters the volatile metabolome of isolates belonging to ST258. Box plots depict relative compound abundance for metabolites that were: (1) identified as discriminatory between ST258 and non-ST258 by RF, linear SVM, and PLS-DA, and (2) significantly different in relative compound abundance between wild-type ST258 isolates and cured ST258 isolates (*p* < 0.05 after Benjamini-Hochberg correction). Red: ST258 WT; purple: ST258 cured; blue: non-CP (all STs); dark red: CP isolates (all other STs). The y-axis (in arbitrary units [a.u.]) corresponds to relative compound abundance, as measured by total ion chromatogram (TIC), following log_10_-transformation, mean-centering, and unit-scaling.

We sought to determine the effect of plasmid curing on the volatile metabolic profile of *K*. *pneumoniae* isolates belonging to ST258. Three CP *K*. *pneumoniae* isolates were successfully cured of their *bla*_KPC_-encoding plasmids (see Materials and Methods), rendering them both phenotypically meropenem-susceptible as well as PCR-negative for the *bla*_KPC_ gene, and plasmid loss was visualized using gel electrophoresis. The parental strains from which we derived these cured isolates all belonged to ST258, and we therefore focused our comparative analysis on the 52 metabolites identified as discriminatory between ST258 and non-ST258 by all three machine learning algorithms, as described in the previous section. Twenty of these metabolites differed significantly (*p* < 0.05 after Benjamini-Hochberg correction) in relative abundance between wild-type CP ST258 isolates and the corresponding, cured non-CP ST258 isolates. Eight of these 20 differentially-abundant metabolites could be assigned either putative compound identifications or compound class identifications, namely: 1-methyl-4-(1-methylethenyl)-cyclohexene, 2-nonanone, 2-undecanone, ethylcyclohexane, *o*-xylene, and three unknown esters (Fig. 6). Plasmid curing further accentuated the difference in relative compound abundance between ST258 and non-ST258 isolates for six of these eight metabolites. For example, 1-methyl-4-(1-methylethenyl)-cyclohexene, 2-nonanone, and 2-undecanone were less abundant in the headspace of ST258 cultures relative to non-ST258 cultures, and plasmid curing resulted in a further decrease in relative abundance. Further, the three unknown esters were more abundant in ST258 cultures relative to non-ST258 cultures at baseline, and plasmid curing resulted in a further increase in relative compound abundance. Further study is necessary to understand the mechanism behind this seemingly paradoxical result.

## Discussion

The present findings suggest that volatile metabolites have utility not only in the detection of high-risk carbapenemase-producing clones (*e*.*g*., *K*. *pneumoniae* ST258), but also in the identification of less commonly-encountered *K*. *pneumoniae* and *E*. *cloacae* STs, such as those that may be specific to a particular institution or geographic region. These volatile metabolic fingerprints were able to distinguish between CP and non-CP isolates of *K*. *pneumoniae* and *E*. *cloacae* with accuracies comparable to those of novel nucleic acid- and matrix-assisted laser desorption/ionization (MALDI)-based approaches, for example^24–27^. For *K*. *pneumoniae*, volatile metabolites could detect CP isolates from four distinct lineages (ST258, ST42, ST307, and one previously-unreported ST). In addition, an ST258-associated signature was identified that was distinct from both non-CP isolates as well as CP isolates belonging to other STs. For *E*. *cloacae*, volatile metabolites could distinguish CP isolates belonging to a single ST (ST171) from non-CP isolates belonging to nine distinct sequence types. In contrast to *K*. *pneumoniae* and *E*. *cloacae*, volatile metabolic fingerprints could not reliably discriminate between CP and non-CP isolates of *E*. *coli* under the experimental conditions employed in this study. For *K*. *pneumoniae* (and to some extent, *E*. *cloacae*), dominant CP lineages are largely distinct from non-CP lineages^22,28–30^, while for *E*. *coli*, the dominant ST131 lineage is commonly encountered in both CP and non-CP variants^14,18^. We therefore suspect that inclusion of ST131 isolates from both CP and non-CP populations may have impacted our ability to discriminate between these two groups. However, we do acknowledge that ST131 isolates could not be differentiated from non-ST131 isolates, irrespective of carbapenem susceptibility, potentially suggesting that this clonal lineage is more metabolically diverse than either ST258 in *K*. *pneumoniae* or ST171 in *E*. *cloacae*.

We observe that CPE produced an abundance of benzene derivatives, esters, and hydrocarbons, while non-CPE produced an abundance of alcohols, aldehydes, heterocycles, and ketones. In bacteria, 2-ketones are well-characterized byproducts of fatty acid metabolism, and aldehydes may arise from numerous processes, including the metabolism of both amino acids and fatty acids^19^. Furthermore, hydrocarbons (*i*.*e*., numerous straight-chain alkanes, branched-chain alkanes, and branched-chain alkenes) have previously been identified as markers of oxidative stress in other environments, such as those produced in the setting of malignancy^31^ and infection^32^. The observation that CPE and non-CPE differ with respect to the production of these specific compound classes suggest that these two groups differ in key aspects of metabolism, including central carbon metabolism (*e*.*g*., the metabolism of carbohydrates, fatty acids, and amino acids) and response to oxidative stress; a notion supported by both prior soluble metabolomic and transcriptomic studies. For example, Low and colleagues demonstrated significantly increased production of both succinate and formate by a largely monophyletic group of CP isolates relative to a broader collection of non-CP isolates^33^. Both of these metabolites are implicated in a broad range of biological processes, with succinate acting as a key player in the citric acid cycle and the metabolism of various amino acids, and formate implicated in the metabolism of pyruvate and folate, as well as the response to oxidative stress^34,35^. Furthermore, Gene Ontology (GO) analysis of transcriptomic data collected by Bruchmann and colleagues^17^ identified 232 biological processes that were significantly enriched in CP isolates belonging to clonal complex 258 (CC258) relative to a diverse collection of non-CP isolates. Some of the most significantly enriched GO terms included “response to stress” (*p* = 7.6 × 10^−7^), “organonitrogen compound catabolic process” (*p* = 4.5 × 10^−8^), and “cellular carbohydrate metabolic process” (*p* = 2.7 × 10^−4^). This soluble metabolomic and transcriptomic data, in combination with our present volatile metabolomic findings, supports the notion that CP and non-CP isolates of *K*. *pneumoniae* differ in fundamental aspects of metabolism.

The ability of volatile metabolites to differentiate between CPE and non-CPE has never previously been assessed, although prior studies have demonstrated the utility of this approach in discriminating between methicillin-resistant and methicillin-susceptible *Staphylococcus* *aureus* (MRSA and MSSA, respectively)^36–39^, and between vancomycin-resistant and vancomycin-susceptible *Enterococcus* (VRE and VSE, respectively)^38^. Like CP *K*. *pneumoniae*, resistant isolates belonging to these species tend to be derived from relatively few clonal lineages^12,13^, which may account, at least in part, for the differential production of volatile metabolites as a function of antibiotic susceptibility. However, prior work involving isogenic strains of MRSA and MSSA have also demonstrated volatile metabolic differences as a function of resistance phenotype^36^, suggesting that the resistance mechanism itself (or genes linked to the resistance mechanism) may also influence the volatile metabolome. The present study thus adds to a growing body of research seeking to identify metabolic signatures capable of identifying antibiotic-resistant bacteria. The identification of such signatures has clinical implications, potentially serving to reduce the “time-to-diagnosis” for bacterial infections (possibly through the development of sensors capable of measuring bacterially-derived volatile metabolites in real time) or monitoring strains with epidemic potential for epidemiological purposes. In addition, this approach also raises fundamental questions about underlying biological differences between resistant and susceptible isolates of the same species, and may provide insight into the tendency of antibiotic resistance to spread via the dissemination of clonal lineages.

In summary, the present study represents a novel approach for the identification of carbapenemase-producing *K*. *pneumoniae* and *E*. *cloacae*, via the analysis of volatile metabolites produced *in vitro*. Our findings indicate that CP and non-CP isolates of both *K*. *pneumoniae* and *E*. *cloacae* can be distinguished from one another with near-perfect accuracy, and that in the case of *K*. *pneumoniae*, the volatile metabolic signature likely includes components that are both related to the genetic lineages from which these isolates are derived, as well as carbapenemase production (or genetic features that are linked to carbapenemase production) itself. The three machine learning algorithms employed in the present study (RF, linear SVM, and PLS-DA) were comparable in performance for most comparisons, although notable variations in model performance were noted in the comparison of CP versus non-CP *K*. *pneumoniae* and ST258 versus non-ST258 *K*. *pneumoniae*. This may reflect some attribute that is specific to the *K*. *pneumoniae* data set (*i*.*e*., highly non-linear data) or may reflect differences in the approaches used by these three algorithms for differentiating between experimental groups. In either case, these results demonstrate the importance of considering multiple statistical approaches when evaluating ‘big data’, such as metabolomics data.

Future studies to more precisely determine the relative contributions of the genetic background versus contributions from carbapenemase production and associated genes should involve the analysis of both resistant and susceptible isolates derived from a single lineage (ideally ST258). Furthermore, the evaluation of the volatile metabolites produced by carbapenem-resistant strains that have acquired resistance via other mechanisms (*e*.*g*., porin mutations combined with extended-spectrum beta-lactamase expression) or other carbapenemases (*e*.*g*., *bla*_OXA_, *bla*_VIM_, or *bla*_IMP_) represents an important future direction. Finally, transcriptomic, proteomic, and/or soluble metabolomic experiments in this area could serve to more-precisely elucidate the biological origins of the volatile metabolic signatures that are capable of distinguishing between CP and non-CP isolates.

## Methods

### Bacterial Strains

Strains consisted of clinical isolates of *K*. *pneumoniae* (n = 60), *E*, *coli* (n = 37), and *E*. *cloacae* (n = 20), a subset of which have been described previously^23,40–42^. Isolates originated from a wide range of clinical sites across the United States, as well as mainland Europe. Multilocus sequence typing (MLST) was performed to assess genetic diversity, as reported previously^43–45^. In short, the loci sequenced for MLST were *gapA*, *infB*, *mdh*, *pgi*, *phoE*, *rpoB*, and *tonB* for *K*. *pneumoniae*, *dnaA*, *fusA*, *gyrB*, *leuS*, *pyrG*, *rplB*, and *rpoB* for *E*. *cloacae*, and *adk*, *fumC*, *gyrB*, *icd*, *mdh*, *purA*, and *recA* for *E*. *coli*. Sequences that differed from known sequences by only a single nucleotide were denoted with a “*”. Novel allelic combinations were denoted with a “***”. Antibiotic susceptibilities had been determined previously for all isolates, utilizing either meropenem or ertapenem to determine carbapenem resistance status, and the presence of *bla*_KPC_ in carbapenem-resistant isolates was confirmed via PCR^46^. Primers and PCR reaction conditions are described in Supplementary Table S2.

### Plasmid Curing

Plasmid curing was achieved by the serial passaging of *bla*_KPC_-expressing *K*. *pneumoniae* on Mueller-Hinton agar (MHA, Becton Dickinson (BD), Franklin Lakes, NJ) followed by replica plating onto MHA containing meropenem (2 μg/mL). All plates were incubated at 37 °C. Colonies that exhibited growth on MHA but did not exhibit growth on MHA containing meropenem were retained for further study. Genotypic loss of *bla*_KPC_ was confirmed via PCR^46^, while phenotypic carbapenem susceptibility was confirmed by an absence of growth on MHA containing meropenem (2 μg/mL).

Plasmid curing was confirmed via gel electrophoresis, using a protocol adapted from Heringa and colleagues^47^. In short, isolates were grown overnight in 25 mL of Bacto^TM^ Brain Heart Infusion broth (Becton Dickinson, Franklin Lakes, NJ) at 37 °C with 200 rpm shaking, and 4 mL were transferred to a 15 mL conical tube, and pelleted by centrifugation (2100 × *g*, 15 min, 4 °C). The supernatant was pipetted off, and the cells were resuspended in 200 μL of resuspension buffer (50 mM dextrose, 10 mM EDTA, and 10 mM Tris-HCl, pH = 8). 400 μL of freshly made lysis buffer (0.2 M NaOH and 1% sodium dodecyl sulfate [SDS]) were added, and the solution was left to incubate for 5 minutes at room temperature. 300 μL each of 7.5 M ammonium acetate and chloroform were added and mixed by inversion. The solution was chilled on ice for 10 minutes and centrifuged (2100 × *g*, 10 min, 4 °C), and 800 μL of the resulting supernatant were added to 200 μL of precipitation solution (30% polyethylene glycol 8000 and 1.5 M NaCl). The tubes were again chilled on ice for 10 minutes, and centrifuged to pellet DNA (18100 × *g*, 5 min, 4 °C). The supernatant was removed, 50 μL of Qiagen buffer EB (Qiagen, Hilden, Germany) were added, and the tubes were incubated at 4 °C for several hours. Plasmid DNA was visualized via agarose gel electrophoresis (0.8% agarose, 40 V, 14 h).

### Culture Conditions and Sample Preparation

Clinical isolates were pre-cultured in Difco^TM^ Mueller-Hinton Broth (MHB, Becton Dickinson (BD), Franklin Lakes, NJ) overnight (5 mL, 37 °C, 200 rpm shaking), diluted 1:1000 into fresh MHB, and incubated for an additional 12 hours (20 mL, 37 °C, 200 rpm shaking). 15 mL of culture were centrifuged (12100 × *g*, 5 min, 4 °C), and 4 mL of supernatant were transferred to a 20 mL air-tight headspace vial that was sealed with a PTFE/silicone cap (Sigma-Aldrich, St. Louis, MO). All cultures were incubated in ice throughout the sample preparation process to quench metabolism, and vials were stored at −20 °C prior to analysis. Three biological replicates were prepared for each isolate, and each biological replicate was analyzed independently.

### Concentration and Analysis of Volatile Metabolites

Headspace volatile metabolites were concentrated and analyzed using headspace solid-phase microextraction two-dimensional gas chromatography time-of-flight mass spectrometry (HS-SPME-GC × GC-TOFMS), as described previously^48–50^. Briefly, headspace metabolites were concentrated using a 2-cm triphasic SPME fiber consisting of divinylbenzene, polydimethylsiloxane, and carboxen (Sigma-Aldrich) and desorbed into the inlet of a Pegasus 4D GC × GC-TOFMS instrument (LECO Corp., St. Joseph, MI) fitted with a two-dimensional column set consisting of a Rxi®-624Sil MS first column followed by a Stabilwax second column, and equipped with a rail autosampler (MPS, Gerstel Inc., Linthicum Heights, MD). Comprehensive HS-SPME-GC × GC-TOFMS parameters are described in Supplementary Table S3.

### Chromatographic Alignment and Data Processing

The Statistical Compare feature of ChromaTOF was used for chromatographic alignment (LECO Corp.). A comprehensive list of parameters used in the alignment of chromatographic data is presented in Supplementary Table S4. Suspected chromatographic artifacts and environmental contaminants were eliminated, as described previously^51^, as were atmospheric gases (CO_2_, Ar, etc.). Peaks eluting prior to 358 s were omitted due to inefficient cryogenic modulation of low molecular weight compounds^52^. Only peaks detected in at least two of three biological replicates were retained for subsequent analyses (“replicate filtering”)^53^. For peaks detected in two of three replicates, small value replacement was used to generate a non-zero value for the third replicate, with one-half of the smallest peak area detected across all chromatograms used as the imputed value^54^. Putative identifications were assigned to metabolites if: (1) a mass spectral match score ≥800 (of 1000) could be identified relative to a compound in the NIST 2011 library, and (2) experimentally-determined retention indices were between the literature values for non-polar and polar column configurations (owing to the mid-polarity of the Rxi624®-Sil stationary phase). Chromatographic data was normalized using Probabilistic Quotient Normalization^55^, and the data were auto-scaled (mean centered and unit scaled) prior to statistical analyses. Inter-replicate variability was assessed after data processing, using Spearman’s rho as the variability measure. For all isolates, the average inter-replicate variability was ≥0.90.

### Statistical analyses

All statistical analyses were performed using R v3.2.2 (R Foundation for Statistical Computing, Vienna, Austria). Data was randomly subdivided into discovery (training) and validation (test) sets 100 times, with approximately two-thirds of samples included in the discovery set (n = 40 *K*. *pneumoniae*, 25 *E*. *coli*, and 13 *E*. *cloacae*), and the remaining one-third in the validation set (n = 20 *K*. *pneumoniae*, 12 *E*. *coli*, and 7 *E*. *cloacae*). To avoid potential over-fitting of the data, biological replicates for a given isolate were included in either the discovery set or the validation set, but never both. Three machine learning algorithms, namely random forest (RF)^56^, linear support vector machines (linear SVM)^57^, and partial least squares-discriminant analysis (PLS-DA)^58^, were used to identify volatile metabolites that were discriminatory between experimental groups (*e*.*g*., carbapenemase-producing versus non-carbapenemase-producing isolates) and predict the class to which validation set samples belonged. Three distinct algorithms were utilized because of differences in their approaches to discriminating between experimental groups and identifying important features.

RF randomly divides samples into a training and a test set, generates a decision tree using the training set samples, and then attempts to classify test set samples using this decision tree. This process is repeated many times, generating a “forest” of trees (500 per iteration of RF in this case). Variable importance, as defined by the mean decrease in accuracy (MDA), is a measure of the difference in classification accuracy between the observed values for a given variable versus randomly permuted values of the same variable. Linear SVM generates a hyperplane using training set samples that optimally separates the experimental groups of interest. SVM is a kernel-based model that involves transformation of data into a higher dimensional space using a kernel function. In the case of linear SVM, this is achieved by obtaining the dot product of the data matrix. Class probabilities for test set samples are proportional to the distance between the test sample and the optimal hyperplane identified using the training set. Variable importance, as defined by feature weights, represents the contribution of each variable to the generation of this optimal hyperplane, with a larger absolute value indicating a greater contribution. Finally, PLS-DA is an algorithm that attempts to maximize the covariance between features and experimental group assignment through the generation of latent variables (LVs). Variable importance, as defined by the variable importance in projection (VIP), is a measure of each variable’s contribution to the PLS model, with a distinct VIP calculated for each LV. For PLS-DA, the optimal number of latent variables was determined via leave-one-out cross-validation of discovery set samples. In all cases, the optimal number of LVs was either one or two.

For all three algorithms, the model was “re-tuned” on the discovery set using only the top discriminatory features (*i*.*e*., the model was re-built using only the discriminatory features), and this re-tuned model was used to predict the class to which validation set samples belonged. The top discriminatory metabolites for an algorithm were identified using the average feature rank across all 100 discovery-validation iterations.

Receiver operating characteristic (ROC) curves were generated using the average validation set class probabilities for each sample, with area under the ROC curve (AUROC) used as a measure of model performance. Youden’s J statistic was used to calculate the class probability threshold that resulted in optimal model sensitivity and specificity for validation set samples^59^. For differences in relative compound abundance, statistical significance was calculated using the Mann-Whitney U-test^60^ with Benjamini-Hochberg correction^61^. RF was performed using the ‘randomForest’ R package, SVM using ‘e1071’, and PLS-DA using ‘mixOmics’. ROC curves were calculated using ‘ROCR’ and bee swarm plots using ‘beeswarm’.

## Electronic supplementary material

Supplementary Material

## Data Availability

The datasets generated during and/or analyzed in the current study are available from the corresponding author on reasonable request.
