# Supplementary material for: Detection of high-risk carbapenem-resistant Klebsiella pneumoniae and Enterobacter cloacae isolates using volatile molecular profiles
Source: Sci Rep. 2018 Sep 5;8:13297. doi: 10.1038/s41598-018-31543-x (PMC6125577; doi:10.1038/s41598-018-31543-x)

Detection of high-risk carbapenem-resistant *Klebsiella pneumoniae* and *Enterobacter cloacae* isolates using volatile molecular profiles

Christiaan A Rees[1], Mavra Nasir[1], Agnieszka Smolinska[2], Alexa E Lewis[3], Katherine R Kane[3], Shannon E Kossmann[3], Orkan Sezer[3], Paola C Zucchi[4], Yohei Doi[5], Elizabeth B Hirsch[6], Jane E Hill[1,7,*]

[1] Geisel School of Medicine, Dartmouth College, Hanover, NH, 03755, United States
[2] Department of Pharmacology and Toxicology, Maastricht University Medical Centre, Maastricht, 6200 MD, The Netherlands
[3] Dartmouth College, Hanover, NH, 03755, United States
[4] Bouvé College of Health Sciences, Northeastern University, Boston, MA, 02115, United States
[5] Division of Infectious Diseases, School of Medicine, University of Pittsburgh, Pittsburgh, PA, 15213, United States
[6] College of Pharmacy, University of Minnesota, Minneapolis, MN, 55455, United States
[7] Thayer School of Engineering, Dartmouth College, Hanover, NH, 03755, United States
[*] Corresponding author (Jane.E.Hill@dartmouth.edu)

Supplementary Information

Supplementary Table S1: Optimal model performances for RF, linear SVM, and PLS-DA.

	**RF**			**Linear SVM**			**PLS-DA**		
**Comparison**	Cut	Sens	Spec	Cut	Sens	Spec	Cut	Sens	Spec
CPE vs. non-CPE (Shared features)	0.515	**0.702**	**0.783**	0.501	**0.684**	**0.717**	0.512	**0.684**	**0.817**
CPE vs. non-CPE (All features)	0.517	**0.839**	**0.869**	0.510	**0.842**	**0.847**	0.488	**0.804**	**0.787**
K. pneumoniae only (KPC+ vs. KPC-)	0.545	**0.929**	**0.938**	0.620	**0.714**	**0.688**	0.486	**0.786**	**0.486**
E. coli only (KPC+ vs. KPC-)	0.486	**0.737**	**0.500**	0.490	**0.842**	**0.500**	0.484	**0.700**	**0.529**
E. cloacae only (KPC+ vs. KPC-)	0.724	**0.900**	**1.000**	0.491	**1.000**	**1.000**	0.542	**0.910**	**1.000**
K. pneumoniae only (ST258 vs. non-ST258)	0.387	**0.895**	**0.951**	0.413	**0.842**	**0.976**	0.432	**0.895**	**0.707**
E. coli only (ST131 vs. non-ST131)	0.569	**0.294**	**0.600**	0.560	**0.353**	**0.650**	0.540	**0.412**	**0.600**

Supplementary Table S2: Primers and PCR reaction conditions.

Species

Enterobacter cloacae

dnaA (50 °C)

dnaA-F: AYA ACC CGC TGT TCC TBT ATG GCG GCA C
dnaA-R: KGC CAG CGC CAT CGC CAT CTG ACG CGG

fusA (50 °C)

fusA-F: TCG CGT TCG TTA ACA AAA TGG ACC GTA T
fusA-R: TCG CCA GAC GGC CCA GAG CCA GAC CCA T
fusA-R(seq): ATC TCT TCA CGY TTG TTA GCG TGC ATC T

gyrB (50 °C)

gyrB-F: TCG ACG AAG CGC TCG CGG GTC ACT GTA A
gyrB-R: GCA GAA CCG CCC GCG GAG TCC CCT TCC A
gyrB-F(seq): AAA ACC GGT ACY ATG GTG CGT TTC TGG
gyrB-R(seq): GCA GAA CCG CCC GCG GAG TCC CCT TCC

leuS (50 °C)

leuS-F: GAT CAR CTS CCG GTK ATC CTG CCG GAA G
leuS-R: ATA GCC GCA ATT GCG GTA TTG AAG GTC T

pyrG (50 °C)

pyrG-F: AYC CBG AYG TBA TTG CRC AYM AGG CGA T
pyrG-R: GCR CGR ATY TCV CCC TSH TCG TCC CAG C

rplB (50 °C)

rplB-F: GTA AAC CGA CAT CTC CGG GTC GTC GCC A
rplB-R: ACC TTT GGT CTG AAC GCC CCA CGG AGT T

rpoB (50 °C)

rpoB-F: CCG AAC CGT TCC GCG AAC ATC GCG CTG G
rpoB-R: CCA GCA GAT CCA GGC TCA GCT CCA TGT T

Escherichia coli

adk (50 °C)

adk-F: ATT CTG CTT GGC GCT CCG GG
adk-R: CCG TCA ACT TTC GCG TAT TT

fumC (50 °C)

fumC-F: TCA CAG GTC GCC AGC GCT TC
fumC-R: GTA CGC AGC GAA AAA GAT TC

gyrB (56 °C)

gyrB-F: TCG GCG ACA CGG ATG ACG GC
gyrB-R: ATC AGG CCT TCA CGC GCA TC

icd (50 °C)

icd-F: ATG GAA AGT AAA GTA GTT GTT CCG GCA CA
icd-R: GGA CGC AGC AGG ATC TGT T

mdh (56 °C)

mdh-F: ATG AAA GTC GCA GTC CTC GGC GCT GCT GGC GG
mdh-R: TTA ACG AAC TCC TGC CCC AGA GCG ATA TCT TTC TT

purA (50 °C)
purA-F: CGC GCT GAT GAA AGA GAT GA
purA-R: CAT ACG GTA AGC CAC GCA GA

recA (56 °C)
recA-F: CGC ATT CGC TTT ACC CTG ACC
recA-R: TCG TCG AAA TCT ACG GAC CGG A

Klebsiella pneumoniae

gapA (60 °C)
gapA-F: TGA AAT ATG ACT CCA CTC ACG G
gapA-R: CTT CAG AAG CGG CTT TGA TGG CTT

infB (50 °C)
infB-F: CTC GCT GCT GGA CTA TAT TCG
infB-R: CGC TTT CAG CTC AAG AAC TTC
infB-F(seq): ACT AAG GTT GCC TCC GGC GAA GC

mdh (50 °C)
mdh-F: CCC AAC TCG CTT CAG GTT CAG
mdh-R: CCG TTT TTC CCC AGC AGC AG

pgi (50 °C)
pgi-F: GAG AAA AAC CTG CCT GTA CTG CTG GC
pgi-R: CGC GCC ACG CTT TAT AGC GGT TAA T
pgi-F(seq): CTG CTG GCG CTG ATC GGC AT
pgi-R(seq): TTA TAG CGG TTA ATC AGG CCG T

phoE (50 °C)
phoE-F: ACC TAC CGC AAC ACC GAC TTC TTC GG
phoE-R: TGA TCA GAA CTG GTA GGT GAT

rpoB (50 °C)
rpoB-F: GGC GAA ATG GCW GAG AAC CA
rpoB-R: GAG TCT TCG AAG TTG TAA CC

tonB (45 °C)
tonB-F: CTT TAT ACC TCG GTA CAT CAG GTT
tonB-R: ATT CGC CGG CTG RGC RGA GAG

PCR reaction conditions

Reagents: 2.5 µL standard Taq reaction buffer (10×), 0.5 µL dNTPs (10 mM), 0.5 µL forward primer (10 µM), 0.5 µL reverse primer (10 µM), 1.0 µL template DNA, 0.125 µL Taq polymerase, 18.975 µL dd H_2O.
Reaction conditions: 95 °C for 30 s (initial denaturation), 95 °C for 30 s (denaturation), (*annealing temperature as specified above*) for 30 s (annealing), 68 °C for 60 s (elongation), repeat denaturation, annealing, and elongation × 34, 68 °C for 5 min (final elongation).

Supplementary Table S3: Instrumental parameters for HS-SPME-GC×GC-TOFMS analysis.

**Headspace solid phase microextraction (HS-SPME)**	
**Fiber length, film thickness, and fiber composition**	2 cm, 50/30 μm, triphasic (divinylbenzene/carboxen/polydimethylsiloxane)
**Headspace exposure conditions**	60 min, 37°C, 200 rpm agitation
**Two-dimensional gas chromatography (GC×GC)**	
**Inlet parameters**	270°C, splitless injection, 180 s desorption time
**Carrier gas and flow rate**	Helium, 2 mL/min (constant flow)
**Column composition and dimensions**	Column 1: Rxi®-624Sil MS, 60 m × 0.25 mm × 1.4 μm*<br>Column 2: Stabilwax, 1 m × 0.25 mm × 0.5 μm*
**GC temperature program**	35°C hold for 30 s,<br>+ 3.5°C/min ramp to 230 °C
**Temperature offsets**	+ 5°C (secondary oven), + 25°C (modulator)
**Modulator parameters**	2 s modulation period, alternating 0.5 s hot/0.5 s cold pulses
**Transfer line temperature**	250°C
**Time-of-flight mass spectrometry (TOFMS)**	
**Acquisition delay**	170 s
**Mass range**	30 - 500 amu
**Acquisition rate**	200 spectra/s
**Detector voltage and voltage offset**	1485 V, + 50 V
**Electron energy**	- 70 V
**Ion source temperature**	200°C

* Dimensions correspond to length × internal diameter × film thickness.

Supplementary Table S4: Alignment parameters for chromatographic data.

Intra-chromatogram data processing	
**Baseline offset**	0.5 (through middle of noise)
**Expected peak areas (1D × 2D)**	12 s × 0.15 s
**Mass spectral match requirement for sub-peak combining**	600 (of 1000)
**Min. S:N for peak identification**	50:1
**Inter-chromatogram data processing**	
**Mass spectral match requirement for peak alignment**	600 (of 1000)
**Max. 1D and 2D RT deviations for peak alignment**	6 s, 0.2 s
**Reduced S:N threshold for peak identification***	10:1
**Min. number of samples necessary for compound reporting**	1

* Peaks identified at a S:N ratio of 50:1 in at least one chromatogram were searched at a reduced threshold of 10:1 in all other chromatograms.

Supplementary Figure S1: Distribution of Enterobacteriaceae isolates by species and carbapenem susceptibilities.

Pie chart depicting the number of isolates of *K. pneumoniae* (red), *E. coli* (orange), and *E. cloacae* (purple) included in the present study, subdivided according to carbapenemase production, with darker colors indicating CP isolates, and lighter colors indicating non-CP isolates.

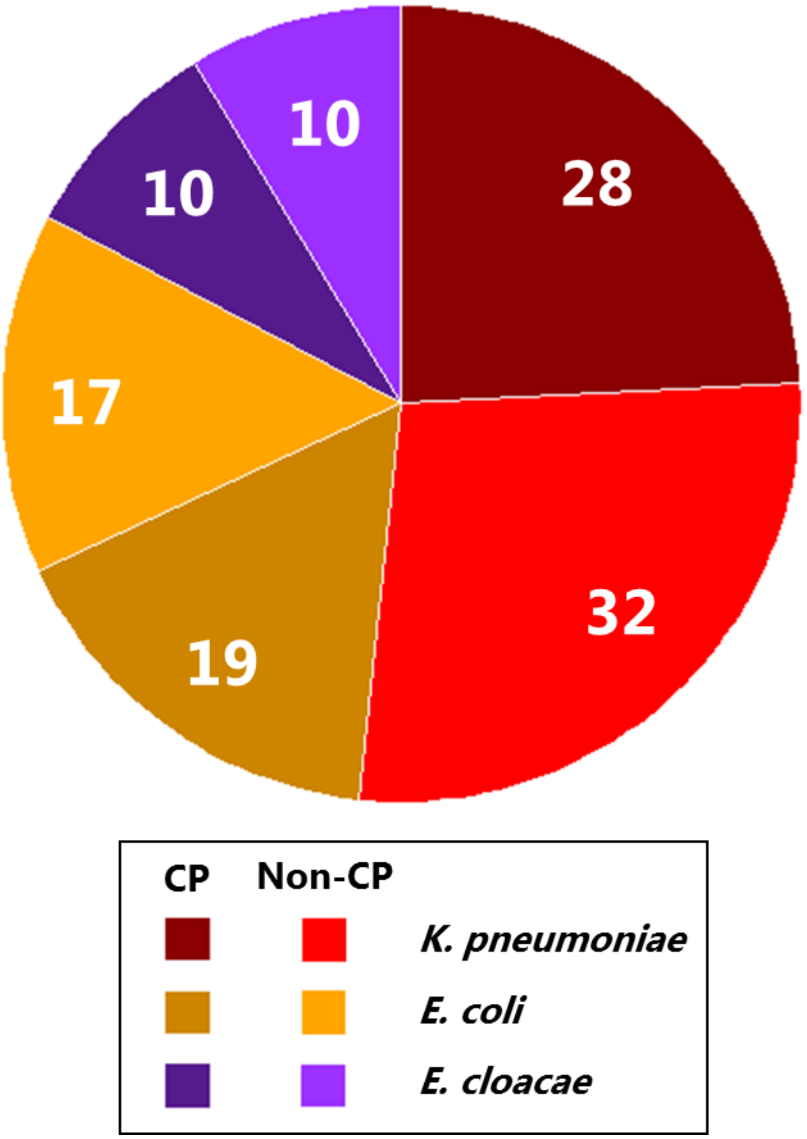

Supplementary Figure S2: MLST distribution for CP and non-CP *K. pneumoniae.*

Bar plot depicting the relative proportions of different sequence types across CP (dark red, *n* = 28) and non-CP (light red, *n* = 32) *K. pneumoniae* isolates. Bars are scaled over the range of 0.0 to 1.0. *** indicates previously unreported STs.

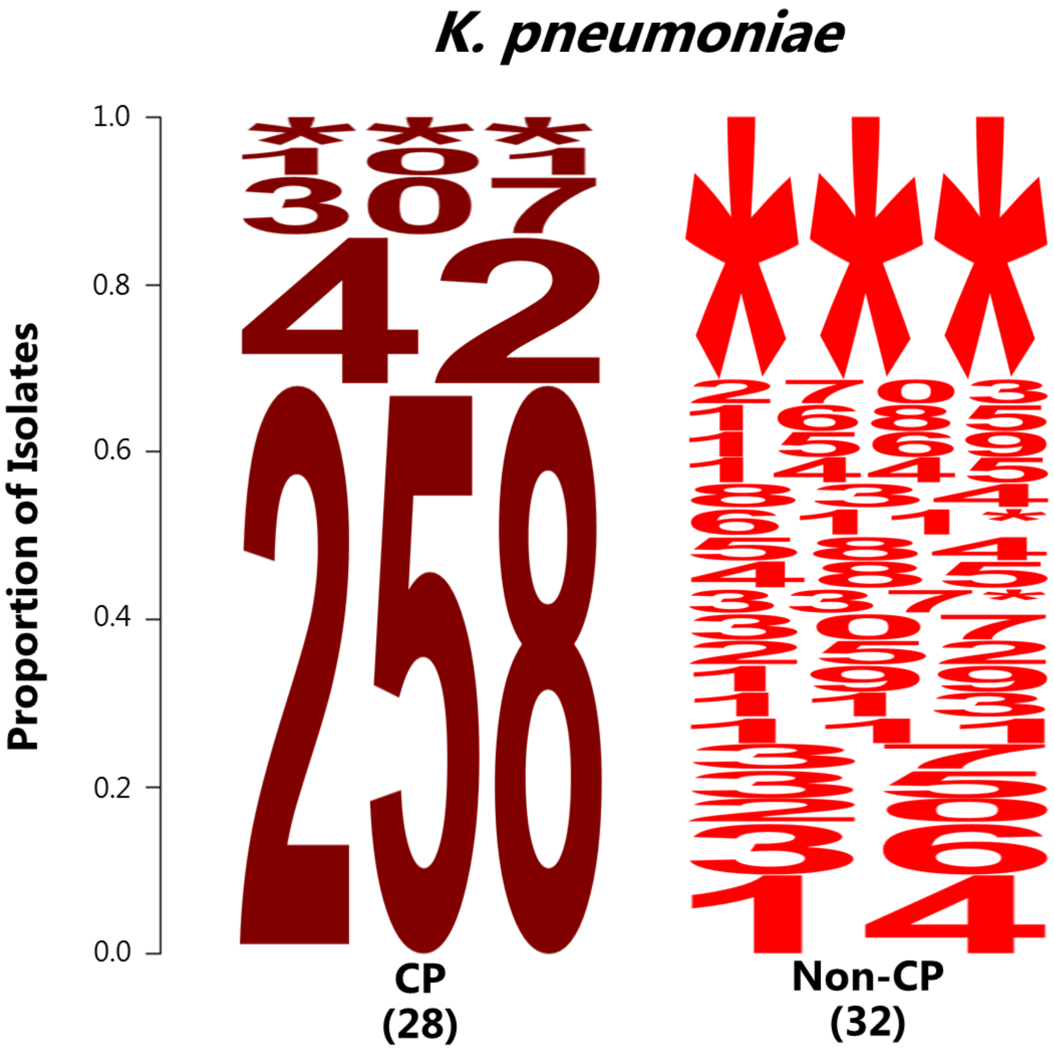

Supplementary Figure S3: MLST distribution for CP and non-CP *E. cloacae.*

Bar plot depicting the relative proportions of different sequence types across CP (dark purple, *n* = 10) and non-CP (light purple, *n* = 10) *E. cloacae* isolates. Bars are scaled over the range of 0.0 to 1.0. *** indicates previously unreported STs.

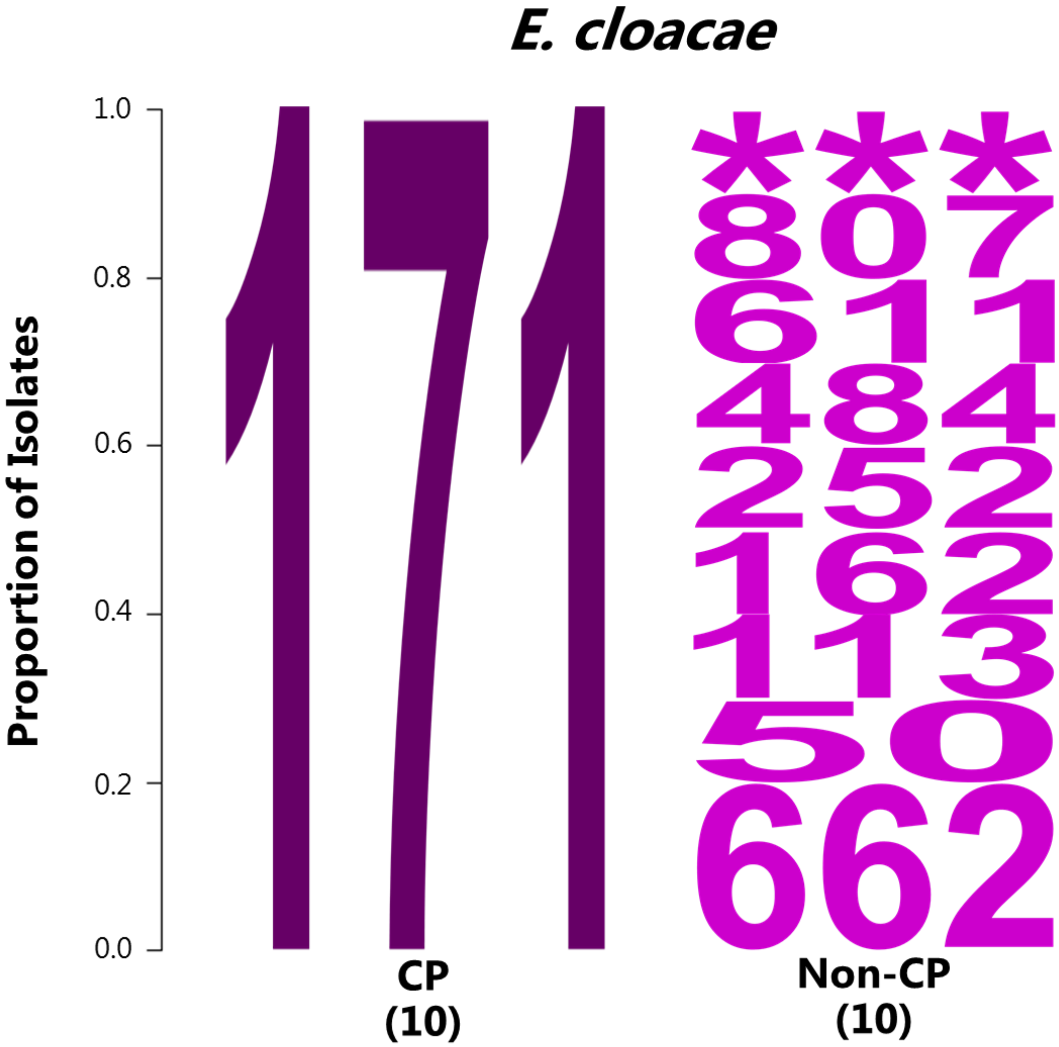

Supplementary Figure S4: MLST distribution for CP and non-CP *E. coli*.

Bar plot depicting the relative proportions of different sequence types across CP (dark orange, n = 19) and non-CP (light orange, n = 18) *E. coli* isolates. Bars are scaled over the range of 0.0 to 1.0.

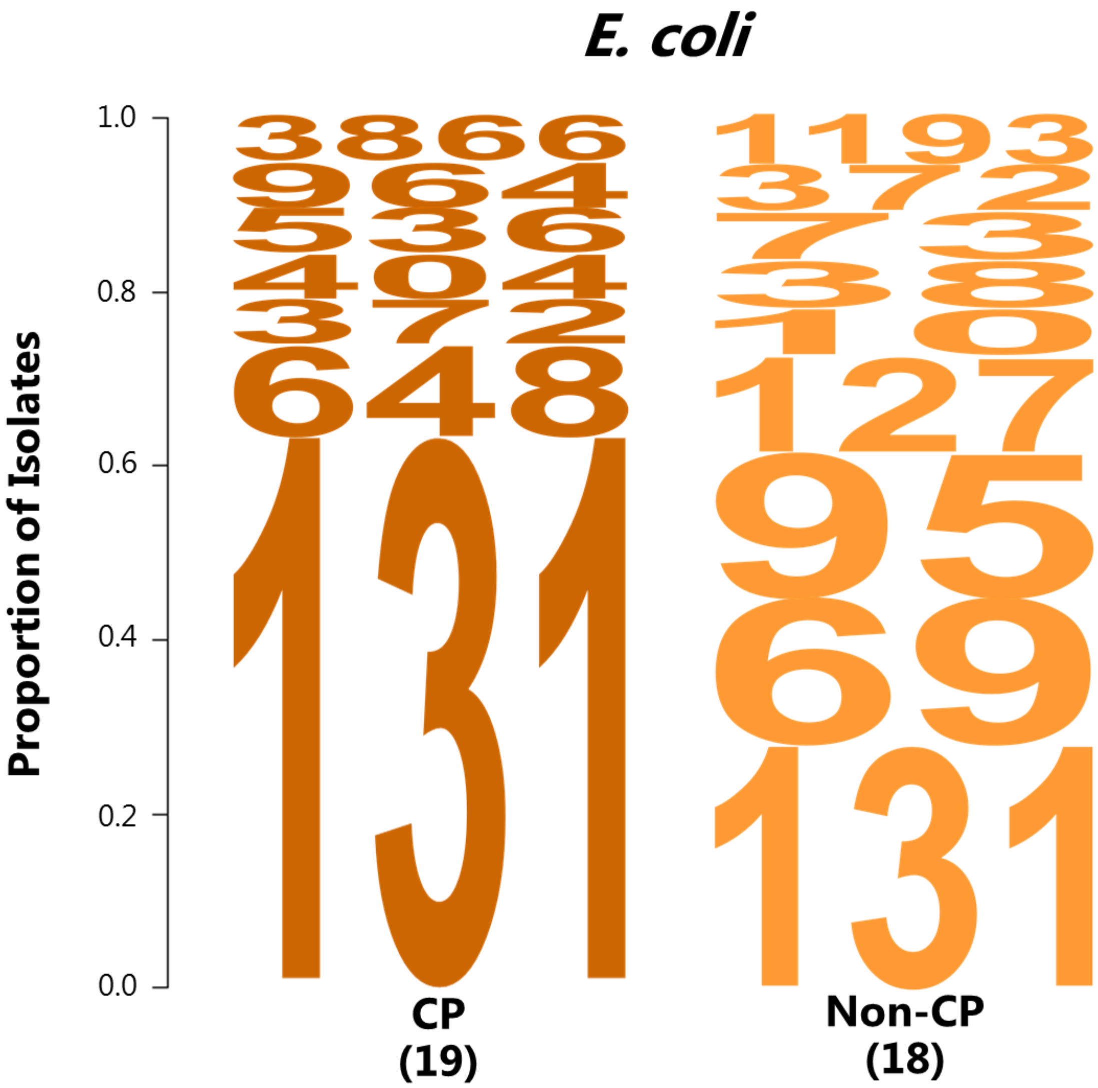

Supplementary Figure S5: Number of top discriminatory metabolites used for the prediction of validation set samples and resultant AUROC.

Line chart depicting the relationship between the number of top discriminatory metabolites used for the prediction of validation set samples (range from 5 to 34) and the resultant AUROC using the top-performing machine learning algorithm for each comparison. Circles: RF; diamonds: linear SVM.

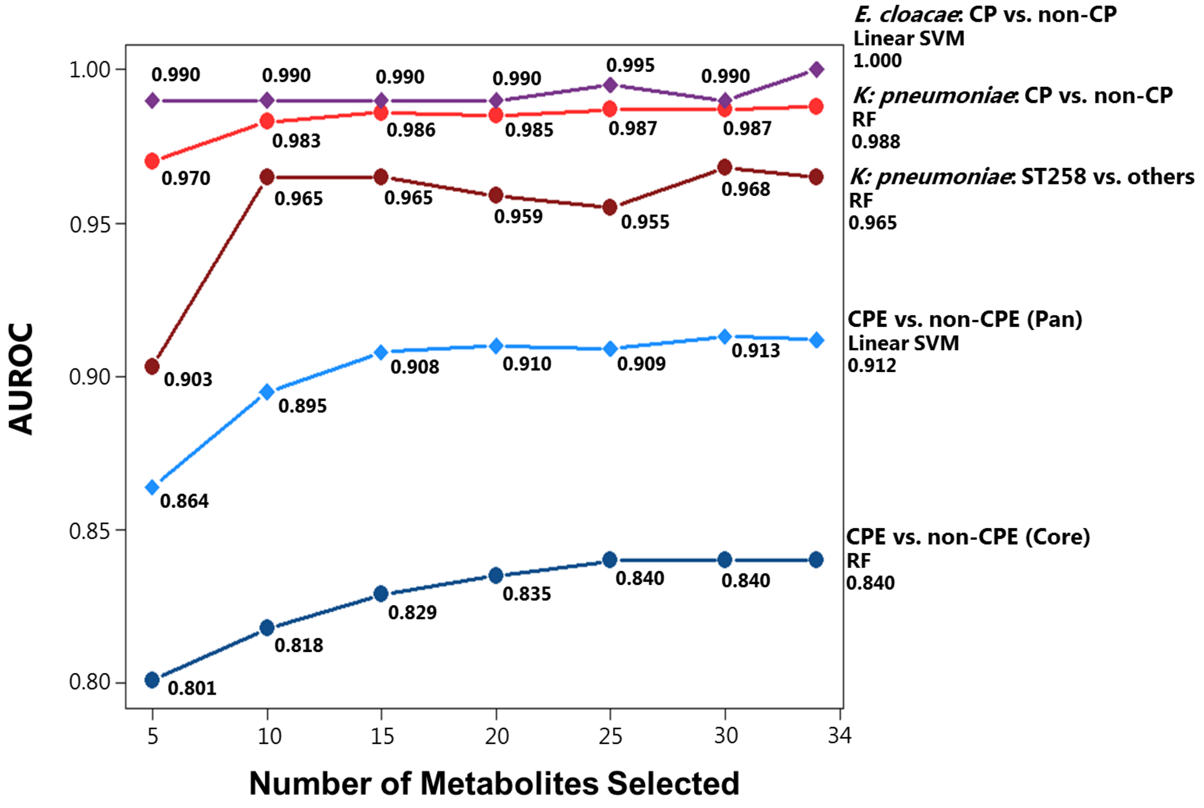

Supplementary Figure S6: Correlation between feature ranks for top discriminatory metabolites of the core metabolome identified using RF, PLS-DA, and linear SVM.

Plots depict feature ranks for metabolites of the core metabolome identified as discriminatory by any of the three machine learning algorithms. Dotted lines correspond to the line of best fit for each comparison, with *r* representing Pearson's correlation coefficient, and *p* representing the *p*-value associated with that correlation.

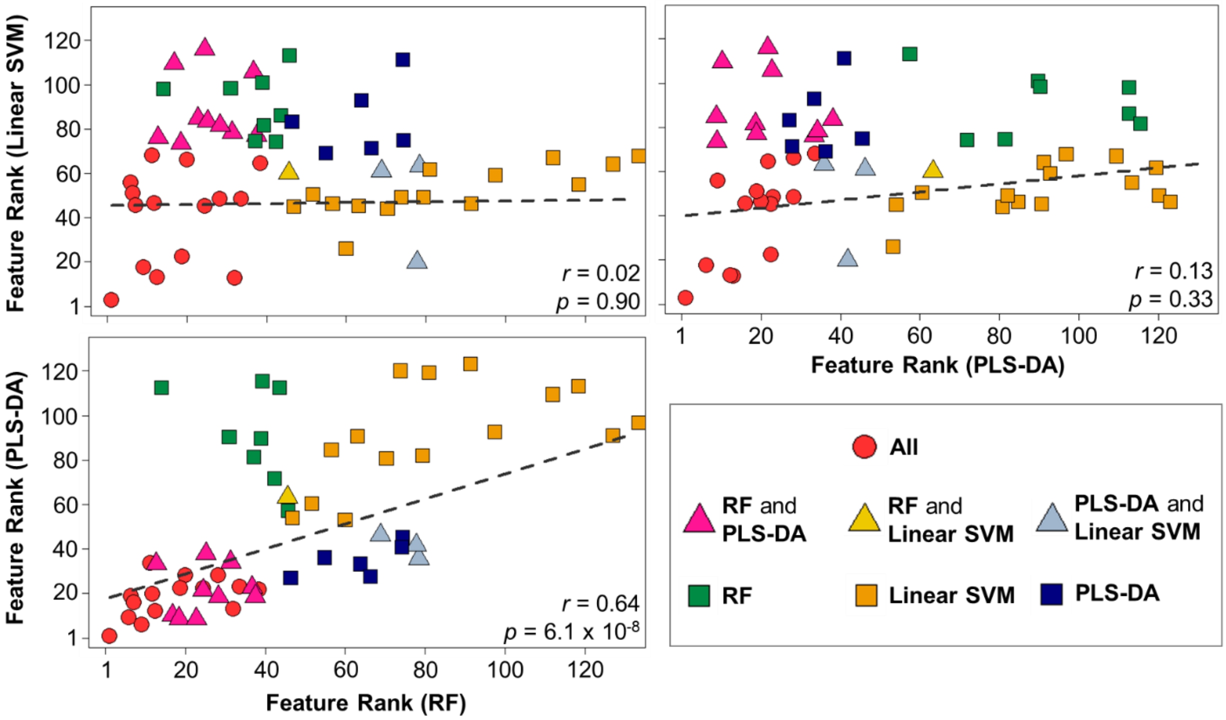

Supplementary Figure S7: Species-level error matrices generated using the core metabolome.

Values are derived from the top-performing machine learning algorithm (RF) using metabolites from the core metabolome. A threshold of 0.500 was used to distinguish correct from incorrect classification, with class probabilities calculated using the average validation set class probabilities for that strain across all discovery-validation splits.

***K. pneumoniae* (88% accuracy)**		Predicted	
		bla$_{KPC}$-positive	*bla*$_{KPC}$-negative
Actual	*bla*$_{KPC}$-positive	24	4
	bla$_{KPC}$-negative	3	29

***E. cloacae* (85% accuracy)**		Predicted	
		bla$_{KPC}$-positive	*bla*$_{KPC}$-negative
Actual	*bla*$_{KPC}$-positive	9	1
	bla$_{KPC}$-negative	2	8

***E. coli* (41% accuracy)**		Predicted	
		bla$_{KPC}$-positive	*bla*$_{KPC}$-negative
Actual	*bla*$_{KPC}$-positive	7	12
	bla$_{KPC}$-negative	10	8

Supplementary Figure S8: Species-level error matrices generated using the pan metabolome. Values are derived from the top-performing machine learning algorithm (linear SVM) using metabolites from the pan metabolome. A threshold of 0.500 was used to distinguish correct from incorrect classification, with class probabilities calculated using the average validation set class probabilities for that strain across all discovery-validation splits.

K. pneumoniae **(93% accuracy)**		Predicted	
		bla$_{KPC}$-positive	*bla*$_{KPC}$-negative
Actual	*bla*$_{KPC}$-positive	27	1
	bla$_{KPC}$-negative	3	29

E. cloacae **(95% accuracy)**		Predicted	
		bla$_{KPC}$-positive	*bla*$_{KPC}$-negative
Actual	*bla*$_{KPC}$-positive	9	1
	bla$_{KPC}$-negative	0	10

E. coli **(65% accuracy)**		Predicted	
		bla$_{KPC}$-positive	*bla*$_{KPC}$-negative
Actual	*bla*$_{KPC}$-positive	12	7
	bla$_{KPC}$-negative	6	12

Supplementary Figure S9: ROC curve for the discrimination between CP and non-CP *E. coli*.

ROC curve is generated using class probabilities for validation set samples, averaged across all 100 discovery-validation splits. Green: RF; orange: linear SVM; blue: PLS-DA. Values in parentheses represent the AUROC associated with each model.

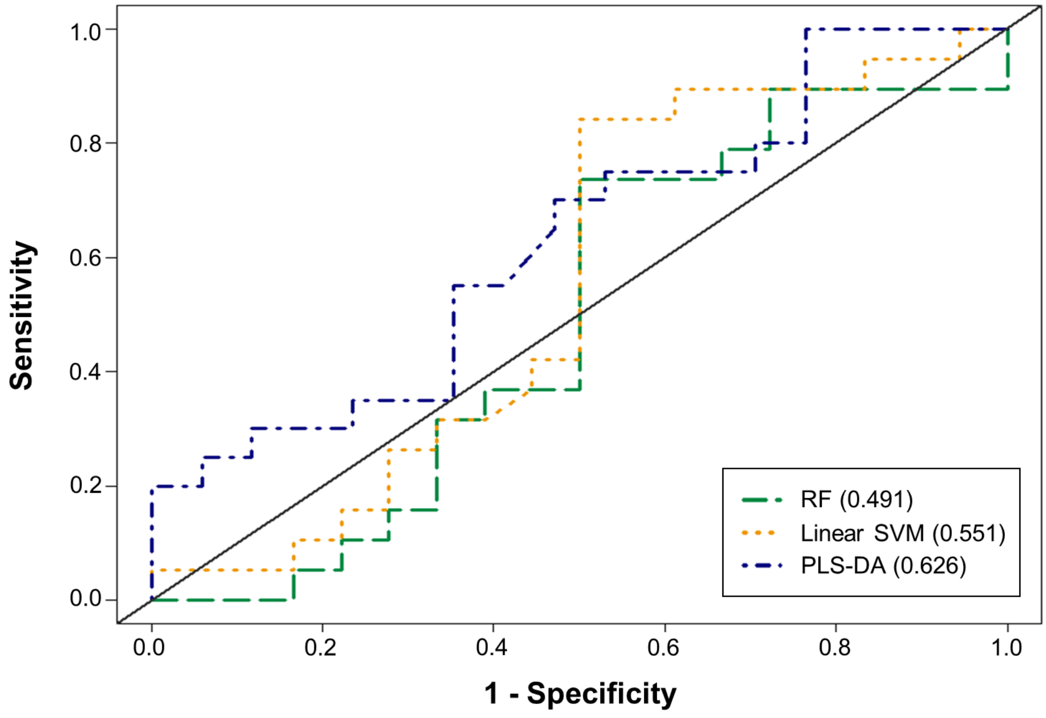

Supplementary Figure S10: Plot depicting class probabilities for CP and non-CP *E. coli*.

Bee swarm plot generated using PLS-DA class probabilities for validation set samples for all 100 discovery-validation splits, separated by ST. Red: CP *E. coli*; blue: non-CP *E. coli*. Points towards the top of the plot have a higher probability of classifying as CP, while those towards the bottom have a higher probability of classifying as non-CP. Horizontal bars represent the median class probability for each ST.

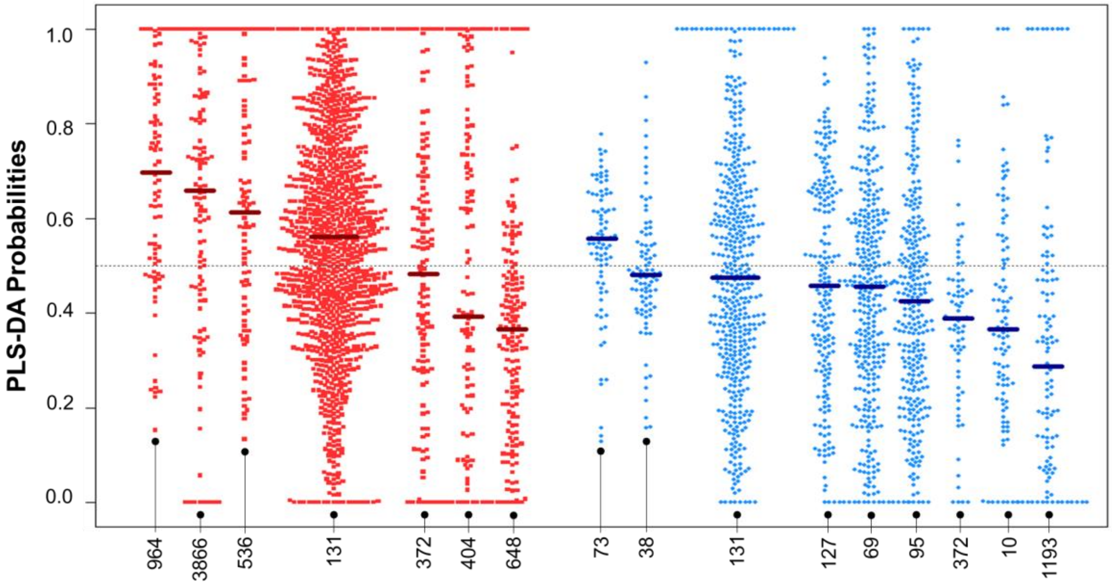

Supplementary Figure S11: ROC curve for the discrimination between ST131 and non-ST131 *E. coli*.

ROC curve is generated using class probabilities for validation set samples, averaged across all 100 discovery-validation splits. Green: RF; orange: linear SVM; blue: PLS-DA. Values in parentheses represent the AUROC associated with each model.

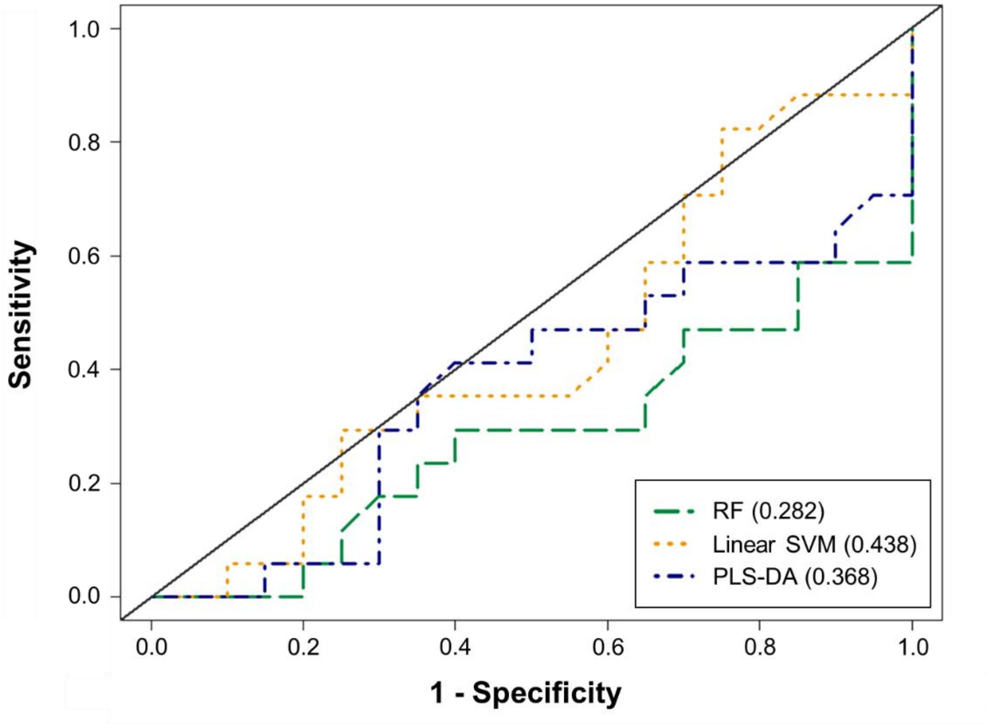

Supplement: Supplementary file 1 — Supplementary Material [file 41598_2018_31543_MOESM1_ESM.pdf]
